# Impact of Pneumococcal and Viral Pneumonia on the Respiratory and Intestinal Tract Microbiomes of Mice

**DOI:** 10.1128/spectrum.03447-22

**Published:** 2023-03-29

**Authors:** Laurin Christopher Gierse, Alexander Meene, Sebastian Skorka, Fabian Cuypers, Surabhi Surabhi, Borja Ferrero-Bordera, Bernd Kreikemeyer, Dörte Becher, Sven Hammerschmidt, Nikolai Siemens, Tim Urich, Katharina Riedel

**Affiliations:** a Institute of Microbiology, University of Greifswald, Greifswald, Germany; b Department of Molecular Genetics and Infection Biology, Institute for Genetics and Functional Genomics, University of Greifswald, Greifswald, Germany; c Institute for Medical Microbiology, Virology and Hygiene, Rostock University Medical Centre, Rostock, Germany; Huazhong University of Science and Technology

**Keywords:** meta-omics, 16S rRNA gene sequencing, metaproteomics, microbiome, influenza A, *Streptococcus pneumoniae*, pneumonia

## Abstract

With 2.56 million deaths worldwide annually, pneumonia is one of the leading causes of death. The most frequent causative pathogens are Streptococcus pneumoniae and influenza A virus. Lately, the interaction between the pathogens, the host, and its microbiome have gained more attention. The microbiome is known to promote the immune response toward pathogens; however, our knowledge on how infections affect the microbiome is still scarce. Here, the impact of colonization and infection with S. pneumoniae and influenza A virus on the structure and function of the respiratory and gastrointestinal microbiomes of mice was investigated. Using a meta-omics approach, we identified specific differences between the bacterial and viral infection. Pneumococcal colonization had minor effects on the taxonomic composition of the respiratory microbiome, while acute infections caused decreased microbial complexity. In contrast, richness was unaffected following H1N1 infection. Within the gastrointestinal microbiome, we found exclusive changes in structure and function, depending on the pathogen. While pneumococcal colonization had no effects on taxonomic composition of the gastrointestinal microbiome, increased abundance of *Akkermansiaceae* and *Spirochaetaceae* as well as decreased amounts of *Clostridiaceae* were exclusively found during invasive S. pneumoniae infection. The presence of *Staphylococcaceae* was specific for viral pneumonia. Investigation of the intestinal microbiomés functional composition revealed reduced expression of flagellin and rubrerythrin and increased levels of ATPase during pneumococcal infection, while increased amounts of acetyl coenzyme A (acetyl-CoA) acetyltransferase and enoyl-CoA transferase were unique after H1N1 infection. In conclusion, identification of specific taxonomic and functional profiles of the respiratory and gastrointestinal microbiome allowed the discrimination between bacterial and viral pneumonia.

**IMPORTANCE** Pneumonia is one of the leading causes of death worldwide. Here, we compared the impact of bacterial- and viral-induced pneumonia on the respiratory and gastrointestinal microbiome. Using a meta-omics approach, we identified specific profiles that allow discrimination between bacterial and viral causative.

## INTRODUCTION

In 2019, pneumonia was ranked as the 2nd leading cause of death in low-income countries and the 4th worldwide (1). Pneumonia can be distinguished as community-acquired pneumonia (CAP), meaning the infection has been acquired outside the hospital setting, and hospital-acquired pneumonia (HAP), which is defined as pneumonia occurring after at least 48 h of hospitalization (2). With nearly 35% of cases in Europe and >25% worldwide, Streptococcus pneumoniae is the predominant cause of CAP, followed by influenza A virus (IAV) (3–9). The risk of acquiring pneumonia is increased in the winter months or by host factors, such as age, comorbidities, and tobacco and/or alcohol consumption (8, 10–13).

As part of the commensal flora of the upper respiratory tract (URT), S. pneumoniae can spread to the lower respiratory tract (12, 14). Colonizing and invasive strains have acquired mechanisms to evade the key innate immune responses, which allows them to exploit and proliferate in their ecological niches (14). S. pneumoniae is able to kill closely related members of the microbiome by production of bacteriocins (15, 16) or to outcompete other microbial families of the URT microbiome by the production of hydrogen peroxide (H_2_O_2_) with the pyruvate oxidase SpxB as a key enzyme (17). Due to these abilities, pneumococci can disrupt the balance of the healthy microbiome, overcome/interrupt the natural colonization barrier, and colonize the URT, a prerequisite to develop CAP (18). This is, for instance, mirrored by an inverse correlation between carriers of pneumococci and staphylococci or a positive correlation of pneumococci with Haemophilus influenzae and Moraxella catarrhalis carriage (19–26). Besides S. pneumoniae, viruses (e.g., IAV) are frequent causes of respiratory disease (27). In contrast to S. pneumoniae, IAVs are obligate pathogens. Under healthy conditions, the URT microbiome has a homeostatic status, and all members are present in optimized proportions (28). When IAVs overcome the natural colonization barrier represented by the microbiome and successfully invade and proliferate in host cells, this balance is disturbed (29–31). The combination of damaged epithelial cells, representing new attachment sites for bacteria (32–35), and alterations in the structure of the microbiome favor outgrowth of opportunistic pathogens, such as Staphylococcus aureus or S. pneumoniae, which ultimately promote secondary bacterial infections (31).

The respiratory and gastrointestinal microbiomes play an important role in disease progression, as it is known that the host’s fitness and immune response can be notably affected by pathogens, medication, or nutrition (36). Furthermore, the microbiome can prevent the initial colonization of pathogenic bacteria (37). The URT microbiome is characterized by high interindividual variability in the community and is influenced by seasonality (20). In the URT microbiome of both adult humans and mice, the phyla (with families shown in parentheses) *Firmicutes* (*Staphylococcaceae*, *Ruminococcaceae*, and *Streptococcaceae*), *Bacteroidetes* (*Prevotellaceae*), *Proteobacteria* (*Pasteurellaceae*), and *Actinobacteria* (*Corynebacteriaceae*, *Propionibacteriaceae*, and *Bifidobacteriaceae*) are predominant (38–40). It is accepted that pathogenesis of disease is often caused by a prior disruption of the nasopharyngeal microbiome, which subsequently causes dysbiosis of the lung microbiome (41). In contrast to the respiratory microbiome, the gastrointestinal tract (GIT) microbiome has been largely explored and is the best-investigated host-associated microbial ecosystem (41). The gastrointestinal tract of the host provides a stable nutrient-rich microenvironment for the microbes, which in turn exert functions like fermentation of dietary fibers and production of metabolites (like short-chain fatty acids [SCFAs]), which are necessary for homeostasis of the immune system (42). At the phylum level, the taxonomic composition of the murine gastrointestinal microbiome resembles the human gastrointestinal microbiome, as both are dominated by *Firmicutes* and *Bacteroidetes*, and around 80 microbial gut genera are shared between mice and humans, even though in different abundances (43). GIT microbes affect the host’s immune response locally in the gut compartment (e.g., barrier functions) and distantly at mucosal interfaces, including the lung (41, 44). Recent studies have shown that the healthy GIT microbiome is crucial for the host’s defense against respiratory infections with influenza A virus and S. pneumoniae by modulating functions of effector immune cells: i.e., alveolar macrophages and neutrophils (29, 45–51). In this context, the gut microbiota was shown to distantly influence pulmonary immunity. This observation is commonly referred to as the gut-lung axis (41, 52). The gut-lung axis was proven to be involved in distant immune modulation during respiratory disease. Therefore, the composition and homeostasis of the GIT microbiome are crucial for maintaining a healthy status of the host.

In the last few decades, murine models have contributed immensely to our improved understanding of disease, as they are by far the most frequently used biomedical models for pneumonia (53). Interactions between the pathogen, the host, and its microbiome are increasingly coming into scientific focus. For instance, monocausal influenza or bacterial superinfection has been proven to contribute to dysbiosis of the intestinal microbiome through altered short-chain fatty acid production (54). However, most recent studies investigated progression of pneumonia or host-microbe interplay or focused on changes in the microbial composition, while the impact on the functional composition of the microbial community itself is far less explored (29, 37–39, 46, 54). Direct comparison of the impact of viral pneumonia and bacterial pneumonia on the respiratory and gastrointestinal microbiomes and their effects on its structure and function is still scarce. Thus, the present study used a meta-omics approach to identify common and pathogen-specific disease-related changes in composition and functionality of the respiratory and gastrointestinal murine microbiome during S. pneumoniae and IAV infections.

## RESULTS AND DISCUSSION

To assess the impact of pneumococcal colonization or pneumococcal/viral pneumonia on the URT and GIT microbiome composition and functionality, an integrated meta-omics approach combining 16S rRNA gene sequencing and metaproteomics was employed. Two infection trials were performed independently from each other. The pneumococcal pneumonia trial harbored three groups (phosphate-buffered saline [PBS]-treated mice, *n* = 10; strain 19F-colonized mice [19F_C], *n* = 10; strain 19F-infected mice with pneumonia [19F_P], *n* = 12), while the setup of viral pneumonia experiments consisted of two groups (PBS, *n* = 6; H1N1, *n* = 12) (see Materials and Methods and Fig. S1 in the supplemental material). Samples for analysis of the URT and GIT microbiome were taken directly after euthanization. Of note, characterization of the respiratory microbiome using metaproteomics was limited and hindered by the low biomass obtained from murine nasal lavage specimens (NALs). In addition, the dominance of high-abundance host proteins and concurrently rare bacterial proteins as well as high salt concentrations hampered a robust meta-proteome analysis of the URT microbiome. Similar challenges have been described in studies of the microbiome from sputum and bronchoalveolar lavage samples in cystic fibrosis or asthma (55, 56). Thus, analysis of the URT microbiome was performed exclusively by 16S rRNA gene sequencing.

### Alterations in the URT microbiome during colonization and infection with S. pneumoniae.

**FIG 1:**
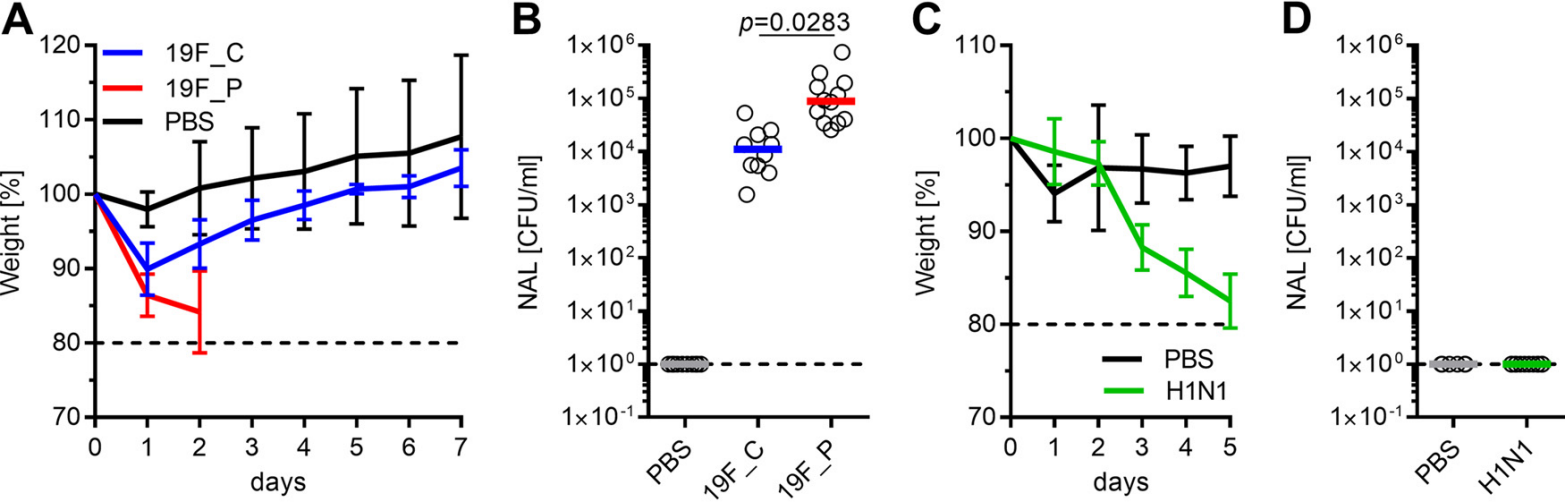
Bacterial and viral infections of C57BL/6J mice. (A) One group of female C57BL/6J mice was intranasally colonized with 1 × 10^7^ CFU S. pneumoniae 19F (19F_C), a second group was infected with 1 × 10^8^ CFU S. pneumoniae 19F (19F_P), and a third group was challenged with PBS as a control. Weight was monitored daily over 7 consecutive days. (B) CFU counts in nasal wash specimens (NAL) at the endpoint of the analyses. (C) C57BL/6J mice were intranasally inoculated with 1 × 10^5^ PFU of H1N1 or challenged with PBS, and weight was monitored daily over 5 consecutive days. (D) To exclude potential bacterial contamination, nasal wash specimens were plated on blood agar. Mean values ± standard deviations (SD) are displayed in panels A and C. Each dot represents one mouse, and horizontal lines display mean values (B and D). Two independent experiments with at least five mice per group were performed: total, for panels A and B, PBS, *n* = 10, 19F_C, *n* = 10, and 19F_P, *n* = 12; total for panels C and D, PBS, *n* = 6, and H1N1, *n* = 12. The level of significance between the groups was determined using Kruskal-Wallis test with Dunn’s multiple-comparison posttest.

**FIG 2:**
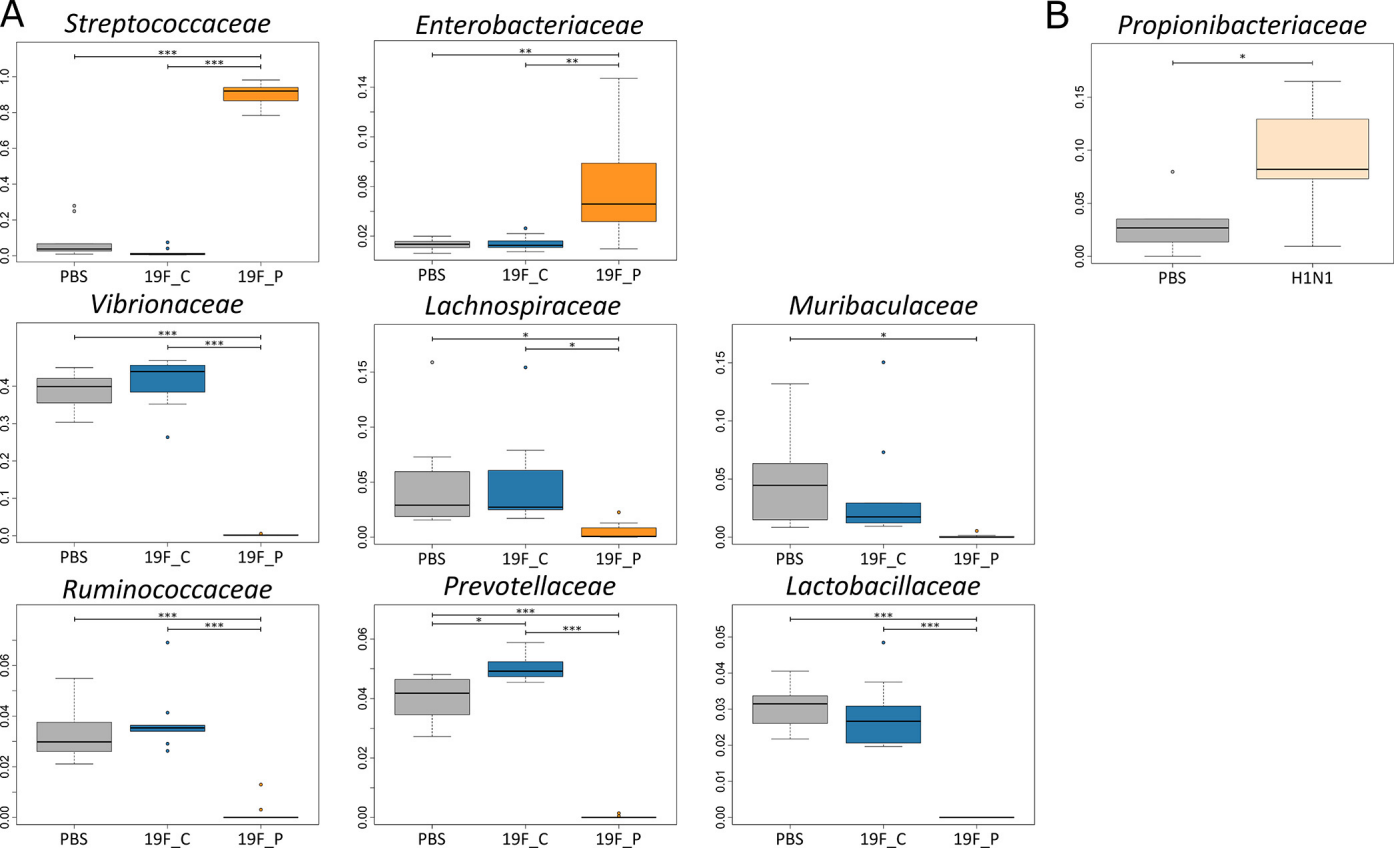
(A) Impact on the taxonomic composition of the URT microbiome of C57BL/6J mice during S. pneumoniae 19F colonization and infection (PBS, *n* = 10; 19F_C, *n* = 10; 19F_P, *n* = 12). (B) Alterations in composition of the respiratory microbiome after IAV infection (PBS, *n* = 4; H1N1, *n* = 12). For better illustration, only high-abundance families (relative abundance > 0.01) with significant changes in relative abundance are shown (ANOVA; *P* = 0.05).

**FIG 3:**
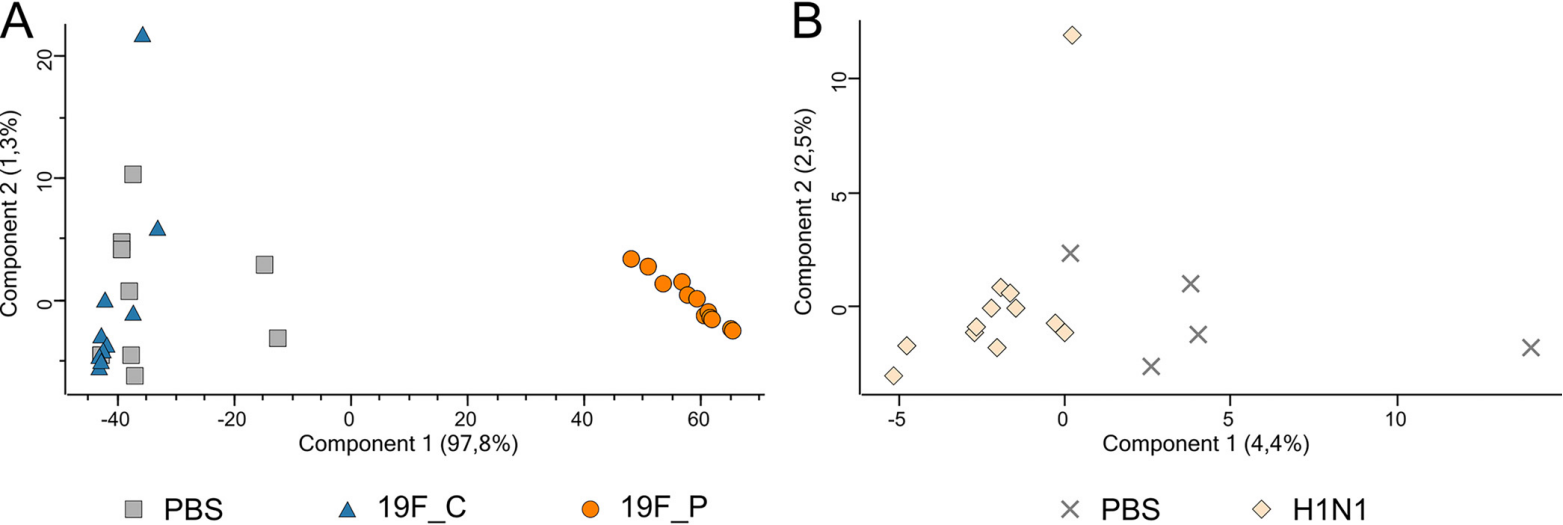
Effects on the taxonomic composition of the URT microbiome of S. pneumoniae 19F colonization (19F_C) or infection (19F_P) (A) and influence of viral pneumonia (B), illustrated in a PCA plot. Shown are results for PBS-treated controls (gray crosses and gray squares), S. pneumoniae-colonized (blue triangles) and -infected (orange circles) mice, and H1N1-infected mice (light orange diamonds).

Intranasal application with a low infection dose (1 × 10^7^ CFU) of pneumococcal strain 19F (19F_C) resulted in an asymptomatic colonization of mice for at least 16 consecutive days. Classification of this infection dose as low was previously verified by Cuypers and colleagues, who established this mouse model to investigate the innate immune response during bacterial and viral coinfection (57). Initial colonization of mice was characterized by a drop in weight on day 1 post-bacterial inoculation (Fig. 1A). The weight normalized in colonized mice over the next days. Seven days after initial colonization, animals were euthanized and cecum content as well as nasal washes were sampled. Carriage of S. pneumoniae was confirmed by plating NALs on blood agar (Fig. 1B). Analysis of the URT microbiome using 16S rRNA gene sequencing revealed an average of 317 (±39) amplicon sequence variants (ASVs) within the URT of healthy mice. Similarly, 322 (±45) ASVs were detected in the URT microbiome of 19F_C mice (see Table S2 in the supplemental material), suggesting that colonization did not affect the number of ASVs, which would be an indication for dysbiosis. Comparison of taxonomic profiles of healthy and S. pneumoniae-colonized mice revealed that the composition of the URT microbiome was nearly unaffected by pneumococcal colonization (Pearson correlation = 0.99) (Fig. 2). The families *Vibrionaceae*, *Streptococcaceae*, *Muribaculaceae*, *Lachnospiraceae*, *Prevotellaceae*, *Ruminococcaceae*, and *Lactobacillaceae* were identified as predominant in the URT microbiome of healthy and 19F_C mice, and only *Prevotellaceae* were significantly increased during pneumococcal colonization. The finding of minor effects of pneumococcal colonization on taxonomic composition was reflected by the principal-component analysis (PCA) plot, showing no separation between the clusters of the healthy and S. pneumoniae-colonized mice (Fig. 3A), and is in accordance with other studies investigating the effects of pneumococcal colonization on the respiratory microbiome (58). Moreover, clinical scoring revealed that the 19F_C group recovered after bacterial colonization and was comparably to the healthy animals. Cuypers and colleagues defined colonization as stable bacterial load in the URT and absence of local and systemic inflammation. Using the same experimental setup, they demonstrated that pneumococcal colonization was locally and systemically asymptomatic at sampling day 7 (57). Asymptomatic carriage of low-abundance pneumococci as part of the commensal flora of the URT microbiome occurs frequently, but development of pneumonia is rare (14). Therefore, our results of a nearly unaffected URT microbiome during bacterial colonization are in line with previous studies.

In contrast to colonization, intranasal infection with a high dose (1 × 10^8^ CFU) of the same strain induced severe pneumonia within 2 days (19F_P) (Fig. 1A), characterized by additional weight loss on day 2 postapplication. Declaration of the infection dose as high was also made by Cuypers and colleagues (57). Animals had to be euthanized due to critical clinical scoring. Analyses of bacterial burden in nasal wash specimens (NALs) showed that all mice harbored pneumococci in the nasopharynx. Higher bacterial counts were detected in NALs of the pneumococcal pneumonia group compared to the group of colonized mice (Fig. 1B). Contrary to the 19F_C group, 16S rRNA gene sequencing revealed that pneumococcal pneumonia reduced the identified ASVs to 27 (±6), while the healthy group contained up to 317 (±39) ASVs (Table S2). A similar observation was made for microbial richness according to Shannon’s H index. Levels of microbial richness were comparable between the PBS control group and the 19F_C mice, while significantly decreased microbial richness was observed in the 19F_P group (Fig. S2A). As expected, *Streptococcaceae* were significantly elevated in the 19F_P cohort, displacing most of the other bacterial families. This observation is in good accordance with the decreased ASVs, microbial richness, and reduced Pearson correlation of 0.18 between the healthy and 19F_P group. Taxonomic profiling showed that *Enterobacteriaceae* was the only family (above 1% relative abundance) that increased in relative abundance in the URT microbiome during pneumococcal pneumonia, while all other predominant families were decreased (Fig. 2). Since a healthy and diverse URT microbiome has been shown to suppress virulence of pathogens (59), decreased α diversity is considered an important step reflecting dysbiosis along the respiratory microbiome (60–62). Therefore, the observed dysbiosis in the URT microbiome can be linked to the overgrowth of S. pneumoniae in the ecological niche, which is a key to pneumococcal disease (63). To further characterize the impact of pneumococcal pneumonia on the URT microbiome, principal-component analysis (PCA) was performed. While the clusters of the PBS control and 19F_C group were nearly identical, the 19F_P group clusters at a distance from the other groups (Fig. 3A). This finding supports the hypothesis of the dysbiosis within the URT microbiome during pneumococcal pneumonia. Of note, scattering within the individual groups was observed. This can be explained by the fact that the respiratory microbiome itself is known to be highly variable (20). In accordance, scattering was reduced in the 19F_P group, which might be explained by the reduced α diversity.

### Effects of H1N1 infection on the URT microbiome.

Similar to pneumococcal infection, viral application of 1 × 10^5^ PFU of non-mouse-adapted H1N1 resulted in a continues disease progression (57). In accordance with the previously published results, H1N1 infection of mice caused continues weight loss, reaching critical clinical scoring 5 days after infection (Fig. 1C). Animals were euthanized, and samples from the respiratory and gastrointestinal tract were taken. To exclude potential bacterial contamination of the nasopharynx, NALs from these animals were plated on blood agar. All animals were negative for pneumococci (Fig. 1D). With an average of 165 (±65) ASVs, IAV-infected mice had slightly increased counts of ASVs compared to the PBS control group (120 ± 85 ASVs) (Table S2). Nevertheless, this increase was not significant. Unlike pneumococcal pneumonia, the viral infection did not reduce microbial richness, reflected by Shannon’s H index (Fig. S2B), and caused only minor changes in the taxonomic profile of the URT (Pearson correlation = 0.97). Analysis of the composition of the URT microbiome identified *Pseudomonadaceae*, *Enterobacteriaceae*, *Lachnospiraceae*, *Lactobacillaceae*, *Staphylococcaceae*, *Propionibacteraceae*, and *Muribaculaceae* as the predominant families. Only *Propionibacteriaceae* showed significantly increased abundance in response to H1N1 infection. The PCA plot showed cluster formation, enabling us to distinguish healthy and H1N1-infected animals (Fig. 3B). Nevertheless, the distance between the groups was not as vast as that observed during pneumococcal pneumonia. In line with our findings, Handa and colleagues described that an infection with influenza caused only mild effects on the respiratory microbiome of humans, while significant changes in the GIT microbiome were detected (64). Thus, we also investigated the impact of pneumonia on the GIT microbiome by an integrated meta-omics approach.

### Pneumonia alters taxonomic composition of the gastrointestinal tract microbiome.

The GIT microbiome was characterized by an integrated meta-omics analysis of cecal content. Results of the taxonomic profiling by independent analysis of the data sets from 16S rRNA gene sequencing and the metaproteomic approach revealed great similarity according to the Pearson correlation of 0.92 (Table S3 and Fig. S3). The taxonomic profile of the gastrointestinal microbiome is based on an average of ~4,650 (±1,280) protein groups (PGs), identified by metaproteomic analysis, that were assigned to 130 families (based on NCBI taxonomy). Taxonomic data of 16S rRNA gene sequencing are available in the supplemental material (Table S4). Most PGs were identified from samples of the 19F_P mice, while the numbers of detected PGs in other samples were significantly smaller (Table S5). The comparably small number of PGs in the PBS-treated mice of the H1N1 infection can be explained by the lower number of animals in this group (*n* = 4 versus *n* = 12). Shannon’s H index revealed that diversity of the microbial community was rather constant, but it was slightly elevated in the GIT microbiome of the 19F_P mice (Table S2 and Fig. S2B). We detected a decreased *Firmicutes*/*Bacteriodetes* ratio during pneumococcal pneumonia, while this ratio increased in animals with H1N1-induced pneumonia.

### Influence of bacterial and viral infection on the GIT microbiome.

The gut microbiome has a protective role during pneumococcal pneumonia, evidenced by increased bacterial dissemination, inflammation, organ failure, and mortality in microbiota-depleted mice (48). Besides its role in local defense against infection, the gastrointestinal microbiome can also modulate immune responses systemically (50, 52, 65–68). In compliance, the GIT microbiome was shown to regulate/mediate immune defense against respiratory tract IAV infection (29, 69) and organ failure in S. pneumoniae-induced sepsis (48).

**FIG 4:**
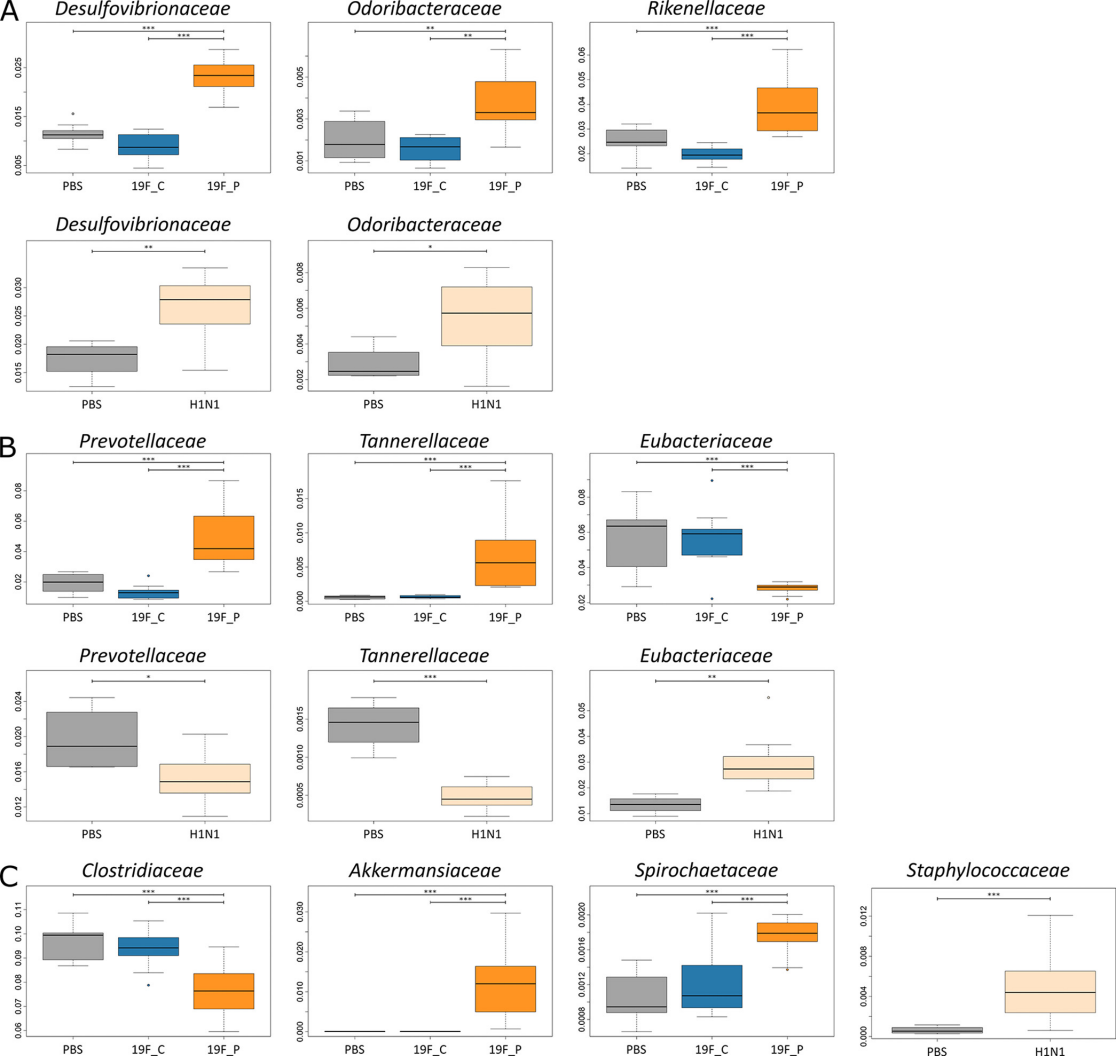
Taxonomic profile of the murine gastrointestinal microbiome highlighting similar and countervailing trends, as well as specific changes due to S. pneumoniae 19F or H1N1 pneumonia, based on metaproteomic data. For better illustration, only higher-abundance families with significant changes are shown (ANOVA; *P* < 0.05). Color code: gray bars, PBS control groups; blue bars, 19F-colonized group; orange bars, 19F-infected group; and light orange, H1N1-infected group. For statistics, see Tables S6A and B.

**FIG 5:**
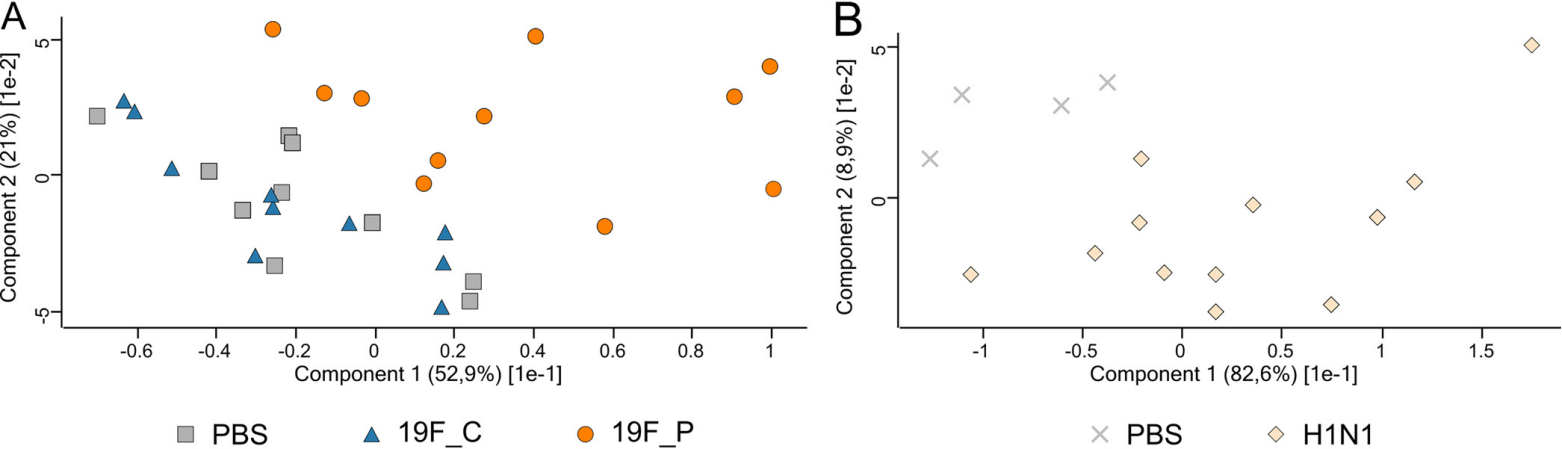
PCA plot illustrating the effects of pneumococcal colonization and mild infection (A), as well as the influence of mild H1N1 infection (B) on the taxonomic composition of the gastrointestinal microbiome. PBS-treated control mice (gray crosses and gray squares), S. pneumoniae-colonized (blue triangles) and -infected (orange circles) mice, and H1N1-infected mice (light orange diamonds).

Similar to the effects on the respiratory microbiome, colonization of the URT with S. pneumoniae 19F had only minor nonsignificant effects on taxonomic structure of the GIT microbiome (Fig. 4). Using the same setup, Cuypers and colleagues demonstrated that colonization of the URT with S. pneumoniae did not have significant effects on the general local inflammatory response 7 days after intranasal inoculation (57). In line with these findings, the observation of no significant changes in taxonomic composition of the GIT microbiome could be linked to the observed minor alterations in the respiratory microbiome. To further characterize the influence of URT colonization by pneumococci on the microbiome, PCA was performed. Healthy and colonized groups clustered together (Fig. 5A). This supports the results of the taxonomic profiling and indicates that pneumococcal colonization of the URT had no effects on the GIT microbiome.

In contrast to the pneumococcal colonization, infection with S. pneumoniae led to several significant changes (analysis of variance [ANOVA]; *P* = 0.05) in the composition of the intestinal microbiome (Table S5). For instance, *Desulfovibrionaceae*, *Rikenellaceae*, *Odoribacteraceae*, *Prevotellaceae*, *Tannerellaceae*, *Akkermansiaceae*, and *Spirochaetaceae* significantly increased in relative abundance in response to pneumococcal pneumonia. On the other hand, *Eubacteriaceae* and *Clostridiaceae* decreased significantly in relative abundance. Of note, significantly increased levels of *Spirochaetaceae* and *Akkermansiaceae*, as well as decreased amounts of *Clostridiaceae*, were exclusively detected in the 19F_P group (Fig. 4C). Reduced food intake is common during severe pneumonia and can be an explanation for rapid weight loss in the 19F_P group (Fig. 1A and C). The reduced nutrient/fiber intake can affect the GIT microbiome (54). For example, competition between gut commensals at the expense of SCFA producers and to the benefit of bacteria using host mucins as energy source have been described (70). In line with this, we found significantly increased abundance of the mucin-degrading family *Akkermansiaceae* and elevated numbers of *Bacteroidaceae* in the GIT microbiome of mice with pneumococcal pneumonia. As the ends of mucin polysaccharides are often equipped with sulfate groups, degradation of mucins leads to the release of these sulfate groups (71). These free sulfate groups can be used as an energy source by sulfate-reducing bacteria. The most dominant family of sulfate reducers in the murine colon are *Desulfovibrionaceae* (72, 73), and indeed, our data indicated that the abundance of *Desulfvibrionaceae* increased during pneumococcal pneumonia. We speculate that increased abundance of *Desulfovibrionaceae* might be linked to a higher level of sulfate caused by mucin degradation. The PCA plot enabled distinction between the PBS, 19F_C, and 19F_P groups. Compared to PCA of the respiratory microbiome, wide scattering within the groups was observed. α diversity and scattering decreased in the URT microbiome during pneumonia, while Shannon’s H index revealed stable microbial richness in the GIT microbiome under all conditions. Thus, scattering within the cohorts could be explained by the dynamic nature of the individual microbiomes.

Similar to the 19F_P mice, the GIT microbiome of H1N1-infected animals exhibited increased levels of *Desulfvibrionaceae* and *Odoribacteraceae*. Moreover, *Prevotellaceae* and *Tannerellaceae* were detected in significantly decreased abundance, while *Eubacteriaceae* significantly increased. Comparison of the compositions of the GIT microbiome during bacterial and viral infection revealed countervailing trends (Fig. 4B): e.g., alterations in abundance of the families *Prevotellaceae*, *Tanerellaceae*, and *Eubacteriaceae*. Most interestingly, our analysis identified *Staphylococcaceae* as the exclusive significantly increased family in the taxonomic profile of the H1N1-infected mice (Fig. 4C). PCA showed separated clusters of the control and H1N1-infected mice, which was similar to the bacterial counterpart (Fig. 5B).

As the composition of the microbiome or the presence of some bacterial species was linked to the host’s health status (74), the exclusively altered bacteria could possibly be used as indicator families for the discrimination between bacterial or viral pneumonia. Langevin and colleagues suggested that microbiome profiling could be used in clinical management, as mild and severe pneumonia caused specific signatures in the microbiome (75). Based on the taxonomic profiles observed in this study, we consider a discrimination between viral and bacterial pneumonia, which could lead to a more target-oriented treatment.

### Pneumococcal and H1N1 infections of the URT shape the functional composition of the GIT microbiome.

As changes in taxonomy favor changes in functional composition, alterations in composition of the GIT microbiome can be expected during pneumonia. Recent studies demonstrated that carbohydrate, energy, and xenobiotic metabolism as well as signal transduction pathways (e.g., SCFA metabolism) of the murine GIT microbiome are affected by pneumococcal as well as viral pneumonia (48, 54, 76). Using the metaproteomic annotation tool PROPHANE 2.0, the average number of 4,650 (±1,280) PGs was assigned to a total number of 1,116 protein descriptions (according to the eggNOG database). Analysis of the functional composition of the GIT microbiome revealed common and specific changes in response to bacterial or viral infection.

### Pneumococcal colonization and infection shape the functional composition of the GIT microbiome.

**FIG 6:**
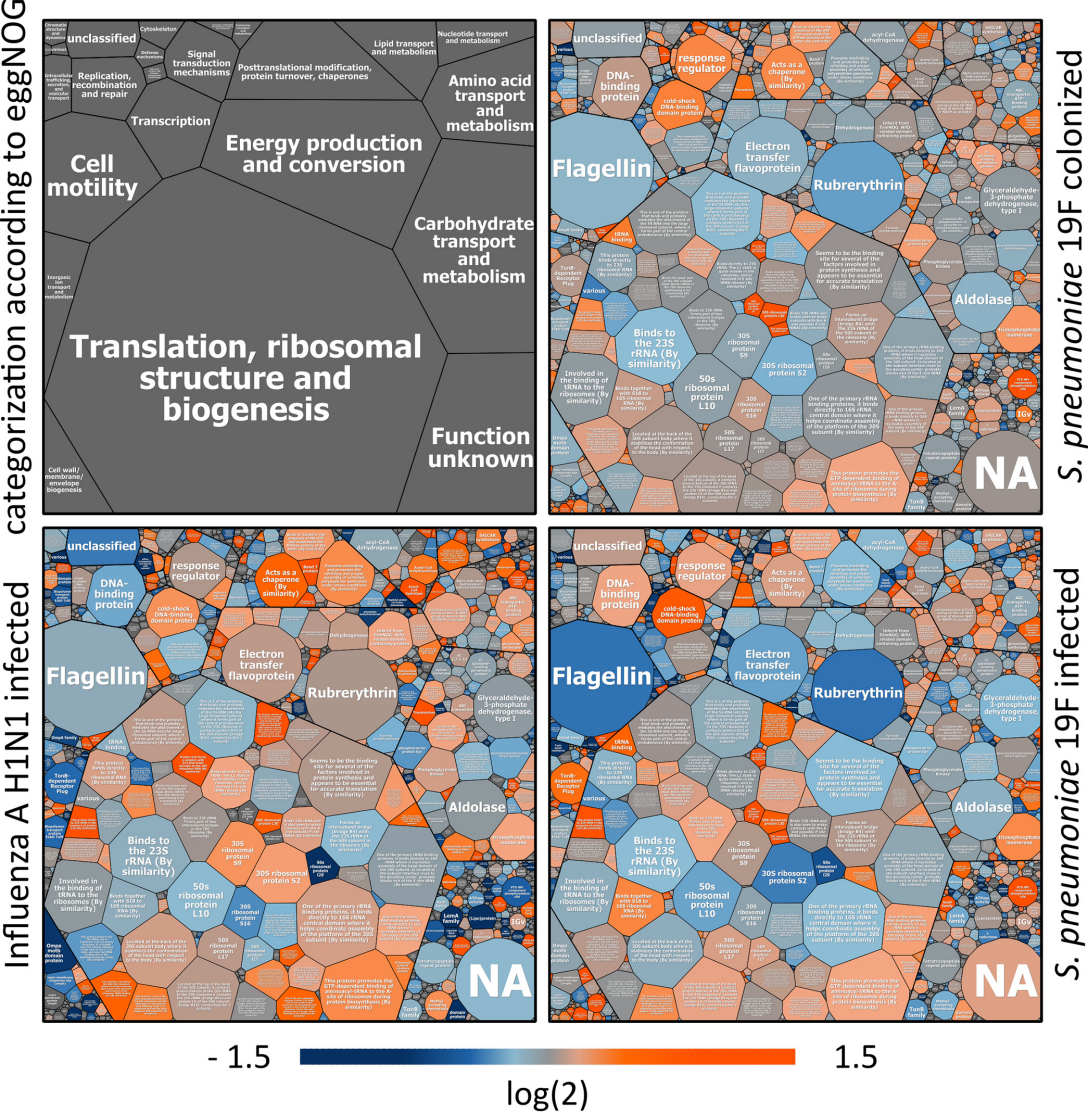
Voronoi treemaps illustrating changes in the functional composition of the murine gastrointestinal microbiome during pneumococcal colonization and infection, as well as H1N1 infection, based on the eggNOG database. The Voronoi treemap on the top left provides an overview of the corresponding functional category of the eggNOG database, to which the individual PGs have been assigned. On the top right, the influence on the gastrointestinal microbiome by pneumococcal colonization of the URT is shown. The Voronoi treemap on the bottom right monitors effects of severe pneumonia on the functional composition of the GIT microbiome, induced by S. pneumoniae infection. The influence of the H1N1 infection is illustrated in the Voronoi treemap on the bottom left. The size of the fields represents the average abundance of the identified PGs under all conditions. For comparison, the log_2_ fold change (FC) (challenged versus healthy) was used. The two-sided gradient orange fields represent an increase in abundance (log_2_ FC value of 1.5), the dark blue fields a decrease in abundance (log_2_ FC value of −1.5), and the gray fields no change in abundance. Higher saturated color indicates more pronounced alterations in the functional composition of the gastrointestinal microbiome.

Our meta-omics analysis revealed that pneumococcal colonization did not affect the GIT microbiome in its taxonomic structure. Nevertheless, alterations in the functional composition were observed. Changes detected in the functional composition of the 19F_C group were similar to the ones we identified in the 19F_P cohort, albeit they were less pronounced in colonized mice (Fig. 6). We assume that distinguishing between colonized and infected mice based on changes in functional profile, as well as identifying specific markers, would be very challenging. Therefore, changes in the functional composition of the microbiome during pneumococcal colonization and infection are discussed together. We observed decreased amounts of protein groups assigned to the functional categories “energy production and conservation.” In particular, expression of proteins from the ATPase complex decreased (Fig. 6). ATPase consists of several subunits and functions as a motor that pumps ions out of the cell or produces ATP (77). It is also involved in the detoxification of metal-thiolate complexes, the most common form of metals in the intracellular space (78). Decreased expression of ATPase might thus lead to accumulation of toxic metal complexes within the members of the GIT microbiome. This relationship between decreased ATPase activity and concomitant increased amounts of secretion proteins was shown as a response of a bacterial community to the exposure of cadmium in a cultivation experiment using two-dimensional (2D) SDS-PAGE (79). Thus, we identified an increased expression of transport and secretion channels assigned to the functional categories “inorganic ion transport and metabolism” and “cell wall/membrane/envelope biogenesis” (e.g., OmpA, TolA, and TonB) correlating with reduced amounts of ATPases, during pneumococcal colonization and infection (Fig. 6). Increased presence of these PGs might contribute to the maintenance of cell homeostasis. Moreover, the expression of electron transfer flavoprotein and rubrerythrin was reduced in the GIT microbiome of mice suffering from pneumococcal pneumonia. Since electron transfer flavoproteins (ETFs) play a central role in transferring electrons from dehydrogenases to the membrane-bound respiratory-chain (80), reduction of ETFs is in accordance with the decreased expression of the ATPase. Our data showed that rubrerythrin, a protein protecting against oxygen-dependent killing by pathogenic anaerobic organisms (81), was mainly produced by members of the phylum *Firmicutes*. Congruent with the lower levels of rubrerythrin in 19F_P mice, we observed a specific reduction of *Firmicutes* in the taxonomic profile of the GIT microbiome during bacterial infection. Congruently, we also observed reduced expression of PGs assigned to the functional category “carbohydrate transport and metabolism” (Fig. 6). The PGs most affected were the enzymes aldolase and glyceraldehyde-3-phosphate dehydrogenase, which were specifically reduced during bacterial pneumonia. Both PGs are involved in ATP production during glycolysis. Thus, this observation is in line with the congruent reduction of PGs assigned to the functional category “energy production and conservation” (i.e., the ATPase complex). Moreover, the abundance of PGs assigned to “cell motility”—i.e., flagellin, the flagellar motor switch protein, and the flagellar proteins FlgE and FliS—was strongly (>2-fold change) reduced specifically in the 19F_P group (Fig. 6). Flagellins are dominant antigens found in members of the *Firmicutes*—for example, in Crohn´s disease (82). Their expression was proved to correlate with the expression of Toll-like receptor 5 (TLR5), which is known to regulate the bacterial load and species composition of *Firmicutes* and *Bacteroidetes* (83, 84). Congruent with this finding, the families *Clostridiaceae*, *Eubacteriaceae*, *Ruminococcaceae*, and *Lachnospiraceae*, were detected in lower abundance exclusively during S. pneumoniae infection (Fig. 4). A decreased number of *Firmicutes* during pneumococcal pneumonia might thus be caused by an immune response toward flagellins.

**FIG 7:**
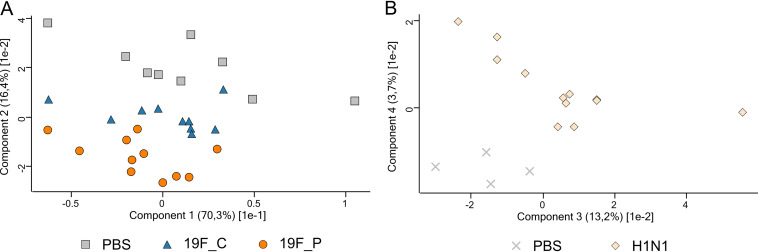
PCA plot illustrating the effects of bacterial and viral pneumonia on the functional assignment of the gastrointestinal microbiome. Shown are results for PBS-treated control mice (gray crosses and gray squares), S. pneumoniae-colonized (blue triangles) and -infected (orange circles) mice, and H1N1-infected mice (light orange diamonds).

PCA demonstrated that the 19F_C and 19F_P cohorts formed clusters, which separated from the PBS control. However, the cluster of the colonized mice was located closer to the PBS control than the cluster of the infected animals (Fig. 7). As changes in taxonomical composition favor changes in the functional structure, separation of the individual clusters was expected. The three clusters showed a distribution from the top right to the bottom left with a great distance between the control and 19F_P cohort. In an intermediate distance from both, the 19F_C cluster was detected. This observation is in line with the observation that colonization caused similar effects on the functional assignments to the 19F_P group, but they were expressed to a lesser extent (Fig. 6).

### H1N1 infection alters functional capacity of the GIT microbiome.

In contrast to the bacterial infection, larger amounts of PGs assigned to “energy production and conversion” were identified during H1N1 infection. Especially, the ATPase complex was significantly (*P* < 0.05) increased in relative abundance. Further examples of PGs that were expressed in countervailing levels in this category were rubrerythrin, electron transfer flavoprotein, rubredoxin, phosphoenolpyruvate carboxykinase, and phosphate butyryltransferase, which were highly abundant during viral infection but decreased during bacterial pneumonia (Fig. 6). Vice versa to the pneumococcal colonization and infection, IAV pneumonia leads to reduced expression of TonB, TolA, and OmpA, PGs belonging to the categories “inorganic ion transport and metabolism” and “cell wall/membrane/envelope biogenesis.” Furthermore, PGs assigned to the categories “carbohydrate metabolism and transport” (i.e., transketolase) and “cell motility” (i.e., FlgE) showed increased expression specifically in H1N1-infected mice (Fig. 6).

In the category “lipid transport and metabolism,” PGs involved in short-chain fatty acid production (i.e., acetyl coenzyme A [acetyl-CoA] acetyltransferase, enoyl-CoA hydratase, and 2-hydroxy-3-oxopropionate reductase) were expressed in larger amounts during viral infection. Moreover, increased expression of lactaldehyde reductase, aldehyde dehydrogenase, phosphate butyryltransferase, acetate kinase, and methylmalonyl-CoA epimerase was observed during pneumococcal and H1N1 pneumonia. Nevertheless, this observation was more pronounced after viral infection (Fig. 6). It is known, that SCFAs produced by members of the GIT microbiome remotely influence the immune response in the lungs and can thereby modulate disease outcome of respiratory infections (46, 85–88). In parallel, increased expression of proteins assigned to amino acid metabolism was detected. Sencio and colleagues confirmed a reduced amount of SCFA in the GIT of H1N1-infected mice, attributed to decreased food intake during viral infection (54). We thus hypothesize that SCFA-producing members of the microbiome seek to synthesize SCFAs from energetically less beneficial sources, such as amino acids, to compensate the reduced production of SCFAs from dietary fibers. Interestingly, observed changes were more distinct during H1N1 pneumonia than in pneumococcal pneumonia.

Using PCA, a distinction between H1N1-infected mice and the corresponding PBS group was possible. Remarkably, the clusters spread over a larger area in the PCA plot (Fig. 7). This finding is similar to the observation of the S. pneumoniae-colonized mice, highlighting minor alterations in the taxonomic composition can cause significant changes in the functional composition of the GIT microbiome.

### Conclusion.

In this study, we compared the amounts of influence of the most common causes of pneumonia, S. pneumoniae and IAV, on the URT and GIT microbiome of mice. Our data revealed that pneumococcal pneumonia resulted in significant changes in the taxonomic and functional profiles of the upper respiratory and gastrointestinal microbiome compared to pneumococcal colonization and IAV infection. Analysis of the taxonomic composition showed that diversity of the URT microbiome decreased during pneumococcal pneumonia but remained unaffected during S. pneumoniae colonization and H1N1 infection. Moreover, using 16S rRNA gene sequencing and metaproteomics for taxonomic profiling, specific taxonomic profiles of the URT and GIT microbiome, depending on the respective pathogen, have been identified. These pathogen-specific changes in the taxonomic profiles of the URT and GIT microbiome allow a discrimination between bacterial and viral pneumonia. In addition to the taxonomic profiling, this study provides new information on the functional response of the GIT microbiome to bacterial and viral pneumonia: i.e., expression changes of proteins involved in energy production, carbohydrate utilization, ion transport, cell motility, and lipid metabolism. More specifically, a reduced expression of ATPase and a concomitant increased expression of transport and secretion channels seemed to be exclusively caused by bacterial pneumonia, whereas an increased expression of enzymes for SCFA production was more frequently found during viral pneumonia. Further studies, addressing the composition of the gastrointestinal and respiratory microbiome as well as the host’s immune response, will provide a more comprehensive picture of the host-microbiome interaction during pneumonia.

## MATERIALS AND METHODS

### Ethics statement.

All animal experiments were carried out in accordance with the recommendations in the *Guide for the Care and Use of Laboratory Animals* (89), the guidelines of the ethics committee at the University of Greifswald and the German regulations of the Society for Laboratory Animal Science (GVSOLAS), and the European Health Law of the Federation of Laboratory Animal Science Associations (FELASA). All experiments were approved by the Landesamt für Landwirtschaft, Lebensmittelsicherheit und Fischerei Mecklenburg-Vorpommern (LALLFV M-V, Rostock, Germany) and the LALLFV M-V ethical board (permit no. 7221.3-1.1-032/17). All efforts were made to minimize suffering, ensure the highest ethical standard and adhere to the 3R principle (reduction, refinement, and replacement).

### Bacterial and viral strains.

Streptococcus pneumoniae 19F (EF3030), a nasopharynx isolate from a child with frequent episodes of otitis media (90, 91), was grown on blood agar plates (Oxoid) and cultivated to mid-log phase (optical density at 600 nm [OD_600_] of 0.35 to 0.40) in Todd-Hewitt broth supplemented with 0.5% (wt/vol) yeast extract (Carl Roth) at 37°C and 5% CO_2_. Influenza virus A/Bavaria/74/2009 (H1N1) was propagated as described by Eisfeld and colleagues (92).

### Mouse infections, monitoring, and sampling.

To characterize the impact of bacterial and viral pneumonia on the respiratory and gastrointestinal microbiome, we used a murine model that had been previously established to investigate the innate immune response during bacterial and viral coinfections (57). In brief, 7-week-old specific-pathogen-free (SPF) female C57BL/6J mice were purchased from Janvier Labs. Mice were maintained within the biosafety level 2 (BSL2) animal facility for 1 week prior to the infection experiments. All diets were provided *ad libitum*. Mouse maintenance complete feed (S8189-S095 for the first 7 weeks and V1535-000 from week 8) (SSNIFF Spezialdiäten GmbH) was used in the animal facility. Randomly assigned groups of 5 to 6 mice (8 weeks old) were intranasally colonized or infected under ketamine/xylazine anesthesia with S. pneumoniae 19F or IAV. For colonization with S. pneumoniae 19F, 20 μL PBS containing 1 × 10^7^ CFU was administered. For pneumonia, 1 × 10^8^ CFU in 20 μL PBS were applied. For viral infections, 42 μL PBS containing 100,000 PFU was used. Control mice were mock treated with an equivalent volume of PBS (Fig. S1). Animals were observed daily for weight and clinical monitoring. At different time points, mice were euthanized and cecum content and nasal wash specimens (NALs) in 1 mL PBS were harvested. Individual cecum content samples were evenly aliquoted to an approximate weight of between 50 and 100 μg, depending in the available weight of the initial sample, and used for 16S rRNA gene sequencing or metaproteomics. Samples were stored until further analyses at −80°C. To quantify bacterial load, NALs were serially diluted and plated on blood agar plates (Oxoid). The provided samples are listed in Table S1 in the supplemental material.

### 16S rRNA gene sequencing and bioinformatic processing.

Nucleic acids of individual cecum content samples were extracted using a bead beating (MP Biomedicals lysing matrix E) phenol-chloroform protocol, followed by nucleic acid precipitation with 3 M Na-acetate and isopropanol. After being washed with 70% (vol/vol) ethanol, DNA pellets were resuspended in diethyl pyrocarbonate (DEPC)-treated Milli-Q water for downstream applications. Nucleic acids of nasal washes were extracted via the Qiagen PowerSoil kit (Qiagen, Hilden, Germany) according to the manufacturer´s protocol. For both sample types, bead beating was performed using the FastPrep-24 5G instrument (MP Biomedicals, Santa Ana, CA, USA) for 45 s at an intensity of 5.5 m/s. Quantification of the DNA content was performed using Qubit double-stranded DNA (dsDNA) broad-range assay kit (Invitrogen) in combination with the Qubit fluorometer 3. Subsequently, DNA extracts were diluted to a final concentration of 5 ng/μL.

For sequence preparation, amplicon and index PCR were performed using the V4 primer pair 515F (5′-GTG-YCA-GCM-GCC-GCG-GTA-A-3′)/806R (5′-GGA-CTA-CNV-GGG-TWT-CTA-AT-3′) with subsequent PCR cleanup between and after both amplifications with AMPure XP beads (93, 94). Quantification of the libraries was done via Invitrogen Qubit dsDNA broad-range assay kit, normalized to a final concentration of 5 pM, denatured with NaOH, and sequenced via Illumina MiSeq with approximate outputs from 30,000 to 130,000 reads per cecum sample and 7,000 to 55,000 reads per sample obtained from nasal washes.

Initial filtering steps as well as truncation and merging of the sequence reads were done using the dada2 pipeline-package v.1.11.1 (R v.3.6.1) in R (95) according to the descriptions in the article by Gierse et al. (96). Amplicon sequence variants (ASVs) were assigned via the SILVA 132 database after removing chimeras, and ASVs identified as chloroplasts and mitochondria were removed. The R packages vegan, ggplot, phyloseq, plyr, and reshape2 were used for advanced bioinformatic processing (e.g., α and β diversities, Bray-Curtis dissimilarities, and nonmetric multidimensional scaling).

### Protein extraction, mass spectrometry, and data analysis.

To extract the total protein amount, a TRIzol-based protocol was applied to the cecal samples following the manufacturer’s instructions. Briefly, 100 mg cecal content was resuspended in 1 mL TRIzol and incubated for 5 min. Afterwards, 0.2 mL chloroform was added and centrifugation was performed (15 min at 12,000 × *g* and 4°C) for separation in the phenol-chloroform, inter, and aqueous phases. Finally, the phenol-chloroform phase was used for protein extraction. To quantify the amount of protein, the Pierce bicinchoninic acid (BCA) protein assay was performed. Afterwards, a 4 to 20% Criterion TGX precast gel (BioRAD, Hercules, CA, USA) was loaded with 30 μg of protein of each sample, run for 1 h at 150 V, and subsequently stained with colloidal Coomassie brilliant blue G-250. After washing of the gel, each lane was cut into 10 pieces, which were further processed to smaller blocks, destained, and tryptic digested. To desalt the peptide-containing solution, ZipTip purification (C_18_ column) (Merck Millipore, Billerica, MA, USA) was performed according to the manufacturer’s protocol. The desalted peptide mixture was vacuum centrifuged until dry. All samples were analyzed by an Easy nLC 1000 coupled online to an Orbitrap Velos mass spectrometer (Thermo Scientific). An 8-μL peptide mixture was loaded into in-house self-packed columns (inside diameter [i.d.], 100 μm; outside diameter [o.d.], 360 μm; length, 200 mm), which were packed with 3.0-μm Dr. Maisch Reprosil C_18_ reversed-phase material (ReproSil-Pur 120 C_18_-AQ), which was first loaded and subsequently eluted with 18 μL of buffer A (0.1% [vol/vol] acetic acid) at a maximum pressure of 22,000,000 Pa. Peptide elution was performed in a nonlinear 100-min gradient of buffer B (0.1% [vol/vol] acetic acid in 99% [vol/vol] acetonitrile) at a constant flow rate of 300 nL/min. Eluted peptides were measured in the Orbitrap with a resolution of *R* = 30,000 with lock-mass correction activated. Following each MS full scan, up to 20 dependent scans were performed in the linear ion trap based on the precursors’ intensity after collision-induced dissociation (CID) fragmentation.

Mass spectrometry data analysis with Mascot Deamon 2.6.2 (Matrix Science, Ltd., London, United Kingdom), Scaffold 4.10.0, X!Tandem (version X!Tandem Alanine 2017.2.1.4), and annotation pipeline Prophane (97) were performed as described by Gierse et al. (96). For statistical analysis, one-way ANOVA with Benjamini-Hochberg correction (*P* = 0.05) and Scheffeé *post hoc* tests were performed. For data visualization, the software tools RStudio (95) and Perseus (98) were used.

### Data availability.

All 16S rRNA gene sequences were submitted to European Nucleotide Archive (ENA) under project no. ERP135841 and accession no. PRJEB51232. The mass spectrometry proteomics data have been deposited in the ProteomeXchange Consortium via the PRIDE (99) partner repository with the data set identifiers PXD036279 (PBS), PXD036129 (19F_C), PXD036115 (19F_P) and, PXD036014 (H1N1).

## References

[1] World Health Organization. 2020. WHO report—the top 10 causes of death. www.who.int/news-room/fact-sheets/detail/the-top-10-causes-of-death. Accessed 3 May 3 2021.

[2] Lanks CW, Musani AI, Hsia DW. 2019. Community-acquired pneumonia and hospital-acquired pneumonia. Med Clin North Am 103:487–501. doi:10.1016/j.mcna.2018.12.008. 30955516

[3] Brown JS. 2012. Community-acquired pneumonia. Clin Med 12:538–543. doi:10.7861/clinmedicine.12-6-538. PMC5922594 23342408

[4] McCullers JA. 2006. Insights into the interaction between influenza virus and pneumococcus. Clin Microbiol Rev 19:571–582. doi:10.1128/CMR.00058-05. 16847087 PMC1539103

[5] Johnstone J, Majumdar SR, Fox JD, Marrie TJ. 2008. Viral infection in adults hospitalized with community-acquired pneumonia: prevalence, pathogens, and presentation. Chest 134:1141–1148. doi:10.1378/chest.08-0888. 18689592 PMC7094572

[6] Prina E, Ranzani OT, Torres A. 2015. Community-acquired pneumonia. Lancet 386:1097–1108. doi:10.1016/S0140-6736(15)60733-4. 26277247 PMC7173092

[7] Said MA, Johnson HL, Nonyane BAS, Deloria-Knoll M, O'Brien KL, Andreo F, Beovic B, Blanco S, Boersma WG, Boulware DR, Butler JC, Carratalà J, Chang F-Y, Charles PGP, Diaz AA, Domínguez J, Ehara N, Endeman H, Falcó V, Falguera M, Fukushima K, Garcia-Vidal C, Genne D, Guchev IA, Gutierrez F, Hernes SS, Hoepelman AIM, Hohenthal U, Johansson N, Kolek V, Kozlov RS, Lauderdale T-L, Mareković I, Masiá M, Matta MA, Miró Ò, Murdoch DR, Nuermberger E, Paolini R, Perelló R, Snijders D, Plečko V, Sordé R, Strålin K, van der Eerden MM, Vila-Corcoles A, Watt JP, AGEDD Adult Pneumococcal Burden Study Team. 2013. Estimating the burden of pneumococcal pneumonia among adults: a systematic review and meta-analysis of diagnostic techniques. PLoS One 8:e60273. doi:10.1371/journal.pone.0060273. 23565216 PMC3615022

[8] Welte T, Torres A, Nathwani D. 2012. Clinical and economic burden of community-acquired pneumonia among adults in Europe. Thorax 67:71–79. doi:10.1136/thx.2009.129502. 20729232

[9] Drijkoningen JJC, Rohde GGU. 2014. Pneumococcal infection in adults: burden of disease. Clin Microbiol Infect 20(Suppl 5):45–51. doi:10.1111/1469-0691.12461. 24313448

[10] Torres A, Peetermans WE, Viegi G, Blasi F. 2013. Risk factors for community-acquired pneumonia in adults in Europe: a literature review. Thorax 68:1057–1065. doi:10.1136/thoraxjnl-2013-204282. 24130229 PMC3812874

[11] van der Poll T, Opal SM. 2009. Pathogenesis, treatment, and prevention of pneumococcal pneumonia. Lancet 374:1543–1556. doi:10.1016/S0140-6736(09)61114-4. 19880020

[12] Blasi F, Mantero M, Santus P, Tarsia P. 2012. Understanding the burden of pneumococcal disease in adults. Clin Microbiol Infect 18(Suppl 5):7–14. doi:10.1111/j.1469-0691.2012.03937.x. 22882668

[13] Cillóniz C, Polverino E, Ewig S, Aliberti S, Gabarrús A, Menéndez R, Mensa J, Blasi F, Torres A. 2013. Impact of age and comorbidity on cause and outcome in community-acquired pneumonia. Chest 144:999–1007. doi:10.1378/chest.13-0062. 23670047

[14] Dockrell DH, Whyte MKB, Mitchell TJ. 2012. Pneumococcal pneumonia: mechanisms of infection and resolution. Chest 142:482–491. doi:10.1378/chest.12-0210. 22871758 PMC3425340

[15] Dawid S, Roche AM, Weiser JN. 2007. The blp bacteriocins of Streptococcus pneumoniae mediate intraspecies competition both in vitro and in vivo. Infect Immun 75:443–451. doi:10.1128/IAI.01775-05. 17074857 PMC1828380

[16] Claverys J-P, Håvarstein LS. 2007. Cannibalism and fratricide: mechanisms and raisons d'être. Nat Rev Microbiol 5:219–229. doi:10.1038/nrmicro1613. 17277796

[17] Regev-Yochay G, Trzcinski K, Thompson CM, Lipsitch M, Malley R. 2007. SpxB is a suicide gene of Streptococcus pneumoniae and confers a selective advantage in an in vivo competitive colonization model. J Bacteriol 189:6532–6539. doi:10.1128/JB.00813-07. 17631628 PMC2045178

[18] Bogaert D, van Belkum A, Sluijter M, Luijendijk A, Groot R, De Rümke HC, Verbrugh HA, Hermans PW. 2004. Colonisation by Streptococcus pneumoniae and Staphylococcus aureus in healthy children. Lancet 363:1871–1872. doi:10.1016/S0140-6736(04)16357-5. 15183627

[19] Chien Y-W, Vidal JE, Grijalva CG, Bozio C, Edwards KM, Williams JV, Griffin MR, Verastegui H, Hartinger SM, Gil AI, Lanata CF, Klugman KP. 2013. Density interactions among Streptococcus pneumoniae, Haemophilus influenzae and Staphylococcus aureus in the nasopharynx of young Peruvian children. Pediatr Infect Dis J 32:72–77. doi:10.1097/INF.0b013e318270d850. 22935873 PMC3525793

[20] Bogaert D, Keijser B, Huse S, Rossen J, Veenhoven R, van Gils E, Bruin J, Montijn R, Bonten M, Sanders E. 2011. Variability and diversity of nasopharyngeal microbiota in children: a metagenomic analysis. PLoS One 6:e17035. doi:10.1371/journal.pone.0017035. 21386965 PMC3046172

[21] Mackenzie GA, Leach AJ, Carapetis JR, Fisher J, Morris PS. 2010. Epidemiology of nasopharyngeal carriage of respiratory bacterial pathogens in children and adults: cross-sectional surveys in a population with high rates of pneumococcal disease. BMC Infect Dis 10:304. doi:10.1186/1471-2334-10-304. 20969800 PMC2974682

[22] Madhi SA, Adrian P, Kuwanda L, Cutland C, Albrich WC, Klugman KP. 2007. Long-term effect of pneumococcal conjugate vaccine on nasopharyngeal colonization by Streptococcus pneumoniae–and associated interactions with Staphylococcus aureus and Haemophilus influenzae colonization–in HIV-infected and HIV-uninfected children. J Infect Dis 196:1662–1666. doi:10.1086/522164. 18008250

[23] Park B, Nizet V, Liu GY. 2008. Role of Staphylococcus aureus catalase in niche competition against Streptococcus pneumoniae. J Bacteriol 190:2275–2278. doi:10.1128/JB.00006-08. 18223076 PMC2293205

[24] Jacoby P, Watson K, Bowman J, Taylor A, Riley TV, Smith DW, Lehmann D, Kalgoorlie Otitis Media Research Project Team. 2007. Modelling the co-occurrence of Streptococcus pneumoniae with other bacterial and viral pathogens in the upper respiratory tract. Vaccine 25:2458–2464. doi:10.1016/j.vaccine.2006.09.020. 17030494 PMC7173051

[25] Pettigrew MM, Gent JF, Revai K, Patel JA, Chonmaitree T. 2008. Microbial interactions during upper respiratory tract infections. Emerg Infect Dis 14:1584–1591. doi:10.3201/eid1410.080119. 18826823 PMC2609881

[26] Regev-Yochay G, Dagan R, Raz M, Carmeli Y, Shainberg B, Derazne E, Rahav G, Rubinstein E. 2004. Association between carriage of Streptococcus pneumoniae and Staphylococcus aureus in children. JAMA 292:716–720. doi:10.1001/jama.292.6.716. 15304469

[27] Taubenberger JK, Kash JC. 2010. Influenza virus evolution, host adaptation, and pandemic formation. Cell Host Microbe 7:440–451. doi:10.1016/j.chom.2010.05.009. 20542248 PMC2892379

[28] Gao Z, Kang Y, Yu J, Ren L. 2014. Human pharyngeal microbiome may play a protective role in respiratory tract infections. Genomics Proteomics Bioinformatics 12:144–150. doi:10.1016/j.gpb.2014.06.001. 24953866 PMC4411333

[29] Ichinohe T, Pang IK, Kumamoto Y, Peaper DR, Ho JH, Murray TS, Iwasaki A. 2011. Microbiota regulates immune defense against respiratory tract influenza A virus infection. Proc Natl Acad Sci USA 108:5354–5359. doi:10.1073/pnas.1019378108. 21402903 PMC3069176

[30] Borges LG, Giongo A, Pereira LD, Trindade FJ, Gregianini TS, Campos FS, Ghedin E, da Veiga AB. 2018. Comparison of the nasopharynx microbiome between influenza and non-influenza cases of severe acute respiratory infections: a pilot study. Health Sci Rep 1:e47. doi:10.1002/hsr2.47. 30623080 PMC6266421

[31] Cauley LS, Vella AT. 2015. Why is coinfection with influenza virus and bacteria so difficult to control? Discov Med 19:33–40. 25636959 PMC4313126

[32] McCullers JA, Webster RG. 2001. A mouse model of dual infection with influenza virus and Streptococcus pneumoniae. Int Congr Ser 1219:601–607. doi:10.1016/S0531-5131(01)00631-8.

[33] McCullers JA, Rehg JE. 2002. Lethal synergism between influenza virus and Streptococcus pneumoniae: characterization of a mouse model and the role of platelet-activating factor receptor. J Infect Dis 186:341–350. doi:10.1086/341462. 12134230

[34] Harford CG, Leidler V, Hara M. 1949. Effect of the lesion due to influenza virus on the resistance of mice to inhaled pneumococci. J Exp Med 89:53–68. doi:10.1084/jem.89.1.53. 18099165 PMC2135848

[35] Francis T, De Torregrosa MV. 1945. Combined infection of mice with H. influenzae and influenza virus by the intranasal route. J Infect Dis 76:70–77. doi:10.1093/infdis/76.1.70.

[36] Kau AL, Ahern PP, Griffin NW, Goodman AL, Gordon JI. 2011. Human nutrition, the gut microbiome and the immune system. Nature 474:327–336. doi:10.1038/nature10213. 21677749 PMC3298082

[37] de Steenhuijsen Piters WAA, Jochems SP, Mitsi E, Rylance J, Pojar S, Nikolaou E, German EL, Holloway M, Carniel BF, Chu MLJN, Arp K, Sanders EAM, Ferreira DM, Bogaert D. 2019. Interaction between the nasal microbiota and S. pneumoniae in the context of live-attenuated influenza vaccine. Nat Commun 10:2981. doi:10.1038/s41467-019-10814-9. 31278315 PMC6611866

[38] Krone CL, Biesbroek G, Trzciński K, Sanders EAM, Bogaert D. 2014. Respiratory microbiota dynamics following Streptococcus pneumoniae acquisition in young and elderly mice. Infect Immun 82:1725–1731. doi:10.1128/IAI.01290-13. 24516113 PMC3993406

[39] Schenck LP, Surette MG, Bowdish DME. 2016. Composition and immunological significance of the upper respiratory tract microbiota. FEBS Lett 590:3705–3720. doi:10.1002/1873-3468.12455. 27730630 PMC7164007

[40] Stearns JC, Davidson CJ, McKeon S, Whelan FJ, Fontes ME, Schryvers AB, Bowdish DME, Kellner JD, Surette MG. 2015. Culture and molecular-based profiles show shifts in bacterial communities of the upper respiratory tract that occur with age. ISME J 9:1246–1259. doi:10.1038/ismej.2014.250. 25575312 PMC4409167

[41] Budden KF, Gellatly SL, Wood DLA, Cooper MA, Morrison M, Hugenholtz P, Hansbro PM. 2017. Emerging pathogenic links between microbiota and the gut-lung axis. Nat Rev Microbiol 15:55–63. doi:10.1038/nrmicro.2016.142. 27694885

[42] Martin-Gallausiaux C, Marinelli L, Blottière HM, Larraufie P, Lapaque N. 2021. SCFA: mechanisms and functional importance in the gut. Proc Nutr Soc 80:37–49. doi:10.1017/S0029665120006916. 32238208

[43] Nguyen TLA, Vieira-Silva S, Liston A, Raes J. 2015. How informative is the mouse for human gut microbiota research? Dis Model Mech 8:1–16. doi:10.1242/dmm.017400. 25561744 PMC4283646

[44] McAleer JP, Kolls JK. 2018. Contributions of the intestinal microbiome in lung immunity. Eur J Immunol 48:39–49. doi:10.1002/eji.201646721. 28776643 PMC5762407

[45] Abt MC, Osborne LC, Monticelli LA, Doering TA, Alenghat T, Sonnenberg GF, Paley MA, Antenus M, Williams KL, Erikson J, Wherry EJ, Artis D. 2012. Commensal bacteria calibrate the activation threshold of innate antiviral immunity. Immunity 37:158–170. doi:10.1016/j.immuni.2012.04.011. 22705104 PMC3679670

[46] Moriyama M, Ichinohe T. 2019. High ambient temperature dampens adaptive immune responses to influenza A virus infection. Proc Natl Acad Sci USA 116:3118–3125. doi:10.1073/pnas.1815029116. 30718396 PMC6386664

[47] Steed AL, Christophi GP, Kaiko GE, Sun L, Goodwin VM, Jain U, Esaulova E, Artyomov MN, Morales DJ, Holtzman MJ, Boon ACM, Lenschow DJ, Stappenbeck TS. 2017. The microbial metabolite desaminotyrosine protects from influenza through type I interferon. Science 357:498–502. doi:10.1126/science.aam5336. 28774928 PMC5753406

[48] Schuijt TJ, Lankelma JM, Scicluna BP, de Sousa e Melo F, Roelofs JJTH, Boer JD, de Hoogendijk AJ, de Beer R, de Vos A, Belzer C, de Vos WM, van der Poll T, Wiersinga WJ. 2016. The gut microbiota plays a protective role in the host defence against pneumococcal pneumonia. Gut 65:575–583. doi:10.1136/gutjnl-2015-309728. 26511795 PMC4819612

[49] Brown RL, Sequeira RP, Clarke TB. 2017. The microbiota protects against respiratory infection via GM-CSF signaling. Nat Commun 8:1512. doi:10.1038/s41467-017-01803-x. 29142211 PMC5688119

[50] Clarke TB, Davis KM, Lysenko ES, Zhou AY, Yu Y, Weiser JN. 2010. Recognition of peptidoglycan from the microbiota by Nod1 enhances systemic innate immunity. Nat Med 16:228–231. doi:10.1038/nm.2087. 20081863 PMC4497535

[51] Bradley KC, Finsterbusch K, Schnepf D, Crotta S, Llorian M, Davidson S, Fuchs SY, Staeheli P, Wack A. 2019. Microbiota-driven tonic interferon signals in lung stromal cells protect from influenza virus infection. Cell Rep 28:245–256.e4. doi:10.1016/j.celrep.2019.05.105. 31269444

[52] Trompette A, Gollwitzer ES, Yadava K, Sichelstiel AK, Sprenger N, Ngom-Bru C, Blanchard C, Junt T, Nicod LP, Harris NL, Marsland BJ. 2014. Gut microbiota metabolism of dietary fiber influences allergic airway disease and hematopoiesis. Nat Med 20:159–166. doi:10.1038/nm.3444. 24390308

[53] Borsa N, Di Pasquale M, Restrepo MI. 2019. Animal models of pneumococcal pneumonia. Int J Mol Sci 20:4220. doi:10.3390/ijms20174220. 31466400 PMC6747103

[54] Sencio V, Barthelemy A, Tavares LP, Machado MG, Soulard D, Cuinat C, Queiroz-Junior CM, Noordine M-L, Salomé-Desnoulez S, Deryuter L, Foligné B, Wahl C, Frisch B, Vieira AT, Paget C, Milligan G, Ulven T, Wolowczuk I, Faveeuw C, Le Goffic R, Thomas M, Ferreira S, Teixeira MM, Trottein F. 2020. Gut dysbiosis during influenza contributes to pulmonary pneumococcal superinfection through altered short-chain fatty acid production. Cell Rep 30:2934–2947.e6. doi:10.1016/j.celrep.2020.02.013. 32130898

[55] Graf AC, Striesow J, Pané-Farré J, Sura T, Wurster M, Lalk M, Pieper DH, Becher D, Kahl BC, Riedel K. 2021. An innovative protocol for metaproteomic analyses of microbial pathogens in cystic fibrosis sputum. Front Cell Infect Microbiol 11:724569. doi:10.3389/fcimb.2021.724569. 34513734 PMC8432295

[56] Houtman R, van den Worm E. 2005. Asthma, the ugly duckling of lung disease proteomics? J Chromatogr B Analyt Technol Biomed Life Sci 815:285–294. doi:10.1016/j.jchromb.2004.08.049. 15652817

[57] Cuypers F, Schäfer A, Skorka SB, Surabhi S, Tölken LA, Paulikat AD, Kohler TP, Otto SA, Mettenleiter TC, Hammerschmidt S, Blohm U, Siemens N. 2021. Innate immune responses at the asymptomatic stage of influenza A viral infections of Streptococcus pneumoniae colonized and non-colonized mice. Sci Rep 11:20609. doi:10.1038/s41598-021-00211-y. 34663857 PMC8523748

[58] Pettigrew MM, Tanner W, Harris AD. 2021. The lung microbiome and pneumonia. J Infect Dis 223 (Suppl 2):S241–S245. doi:10.1093/infdis/jiaa702. 33330898

[59] Wu BG, Segal LN. 2018. The lung microbiome and its role in pneumonia. Clin Chest Med 39:677–689. doi:10.1016/j.ccm.2018.07.003. 30390741 PMC6221463

[60] Kelly BJ, Imai I, Bittinger K, Laughlin A, Fuchs BD, Bushman FD, Collman RG. 2016. Composition and dynamics of the respiratory tract microbiome in intubated patients. Microbiome 4:7. doi:10.1186/s40168-016-0151-8. 26865050 PMC4750361

[61] Dickson RP, Erb-Downward JR, Huffnagle GB. 2014. Towards an ecology of the lung: new conceptual models of pulmonary microbiology and pneumonia pathogenesis. Lancet Respir Med 2:238–246. doi:10.1016/S2213-2600(14)70028-1. 24621685 PMC4004084

[62] Shankar J, Nguyen MH, Crespo MM, Kwak EJ, Lucas SK, McHugh KJ, Mounaud S, Alcorn JF, Pilewski JM, Shigemura N, Kolls JK, Nierman WC, Clancy CJ. 2016. Looking beyond respiratory cultures: microbiome-cytokine signatures of bacterial pneumonia and tracheobronchitis in lung transplant recipients. Am J Transplant 16:1766–1778. doi:10.1111/ajt.13676. 26693965

[63] Bogaert D, de Groot R, Hermans PW. 2004. Streptococcus pneumoniae colonisation: the key to pneumococcal disease. Lancet Infect Dis 4:144–154. doi:10.1016/S1473-3099(04)00938-7. 14998500

[64] Hanada S, Pirzadeh M, Carver KY, Deng JC. 2018. Respiratory viral infection-induced microbiome alterations and secondary bacterial pneumonia. Front Immunol 9:2640. doi:10.3389/fimmu.2018.02640. 30505304 PMC6250824

[65] Schuijt TJ, van der Poll T, de Vos WM, Wiersinga WJ. 2013. The intestinal microbiota and host immune interactions in the critically ill. Trends Microbiol 21:221–229. doi:10.1016/j.tim.2013.02.001. 23454077

[66] Deshmukh HS, Liu Y, Menkiti OR, Mei J, Dai N, O'Leary CE, Oliver PM, Kolls JK, Weiser JN, Worthen GS. 2014. The microbiota regulates neutrophil homeostasis and host resistance to Escherichia coli K1 sepsis in neonatal mice. Nat Med 20:524–530. doi:10.1038/nm.3542. 24747744 PMC4016187

[67] Caballero S, Pamer EG. 2015. Microbiota-mediated inflammation and antimicrobial defense in the intestine. Annu Rev Immunol 33:227–256. doi:10.1146/annurev-immunol-032713-120238. 25581310 PMC4540477

[68] Blaser M, Bork P, Fraser C, Knight R, Wang J. 2013. The microbiome explored: recent insights and future challenges. Nat Rev Microbiol 11:213–217. doi:10.1038/nrmicro2973. 23377500

[69] Wang J, Li F, Sun R, Gao X, Wei H, Li L-J, Tian Z. 2013. Bacterial colonization dampens influenza-mediated acute lung injury via induction of M2 alveolar macrophages. Nat Commun 4:2106. doi:10.1038/ncomms3106. 23820884 PMC3715851

[70] Desai MS, Seekatz AM, Koropatkin NM, Kamada N, Hickey CA, Wolter M, Pudlo NA, Kitamoto S, Terrapon N, Muller A, Young VB, Henrissat B, Wilmes P, Stappenbeck TS, Núñez G, Martens EC. 2016. A dietary fiber-deprived gut microbiota degrades the colonic mucus barrier and enhances pathogen susceptibility. Cell 167:1339–1353.e21. doi:10.1016/j.cell.2016.10.043. 27863247 PMC5131798

[71] Corfield AP, Wagner SA, Clamp JR, Kriaris MS, Hoskins LC. 1992. Mucin degradation in the human colon: production of sialidase, sialate O-acetylesterase, N-acetylneuraminate lyase, arylesterase, and glycosulfatase activities by strains of fecal bacteria. Infect Immun 60:3971–3978. doi:10.1128/iai.60.10.3971-3978.1992. 1398908 PMC257425

[72] Bisson-Boutelliez C, Massin F, Dumas D, Miller N, Lozniewski A. 2010. Desulfovibrio spp. survive within KB cells and modulate inflammatory responses. Mol Oral Microbiol 25:226–235. doi:10.1111/j.2041-1014.2009.00550.x. 20536750

[73] Zhang P, Li L, Han X, Li Q, Zhang X, Liu JJ, Wang Y. 2020. Fecal microbiota transplantation improves metabolism and gut microbiome composition in db/db mice. Acta Pharmacol Sin 41:678–685. doi:10.1038/s41401-019-0330-9. 31937933 PMC7468362

[74] Hills RD, Pontefract BA, Mishcon HR, Black CA, Sutton SC, Theberge CR. 2019. Gut microbiome: profound implications for diet and disease. Nutrients 11:1613. doi:10.3390/nu11071613. 31315227 PMC6682904

[75] Langevin S, Pichon M, Smith E, Morrison J, Bent Z, Green R, Barker K, Solberg O, Gillet Y, Javouhey E, Lina B, Katze MG, Josset L. 2017. Early nasopharyngeal microbial signature associated with severe influenza in children: a retrospective pilot study. J Gen Virol 98:2425–2437. doi:10.1099/jgv.0.000920. 28884664 PMC7011740

[76] Samuelson DR, Charles TP, de La Rua NM, Taylor CM, Blanchard EE, Luo M, Shellito JE, Welsh DA. 2016. Analysis of the intestinal microbial community and inferred functional capacities during the host response to Pneumocystis pneumonia. Exp Lung Res 42:425–439. doi:10.1080/01902148.2016.1258442. 27925857 PMC5304582

[77] Yoshida M, Muneyuki E, Hisabori T. 2001. ATP synthase—a marvellous rotary engine of the cell. Nat Rev Mol Cell Biol 2:669–677. doi:10.1038/35089509. 11533724

[78] Nies DH. 1999. Microbial heavy-metal resistance. Appl Microbiol Biotechnol 51:730–750. doi:10.1007/s002530051457. 10422221

[79] Lacerda CMR, Choe LH, Reardon KF. 2007. Metaproteomic analysis of a bacterial community response to cadmium exposure. J Proteome Res 6:1145–1152. doi:10.1021/pr060477v. 17284062

[80] Toogood HS, Leys D, Scrutton NS. 2007. Dynamics driving function: new insights from electron transferring flavoproteins and partner complexes. FEBS J 274:5481–5504. doi:10.1111/j.1742-4658.2007.06107.x. 17941859

[81] Mydel P, Takahashi Y, Yumoto H, Sztukowska M, Kubica M, Gibson FC, Kurtz DM, Travis J, Collins LV, Nguyen K-A, Genco CA, Potempa J. 2006. Roles of the host oxidative immune response and bacterial antioxidant rubrerythrin during Porphyromonas gingivalis infection. PLoS Pathog 2:e76. doi:10.1371/journal.ppat.0020076. 16895445 PMC1522038

[82] Lodes MJ, Cong Y, Elson CO, Mohamath R, Landers CJ, Targan SR, Fort M, Hershberg RM. 2004. Bacterial flagellin is a dominant antigen in Crohn disease. J Clin Invest 113:1296–1306. doi:10.1172/JCI200420295. 15124021 PMC398429

[83] Schwab C, Berry D, Rauch I, Rennisch I, Ramesmayer J, Hainzl E, Heider S, Decker T, Kenner L, Müller M, Strobl B, Wagner M, Schleper C, Loy A, Urich T. 2014. Longitudinal study of murine microbiota activity and interactions with the host during acute inflammation and recovery. ISME J 8:1101–1114. doi:10.1038/ismej.2013.223. 24401855 PMC3996699

[84] Vijay-Kumar M, Aitken JD, Carvalho FA, Cullender TC, Mwangi S, Srinivasan S, Sitaraman SV, Knight R, Ley RE, Gewirtz AT. 2010. Metabolic syndrome and altered gut microbiota in mice lacking Toll-like receptor 5. Science 328:228–231. doi:10.1126/science.1179721. 20203013 PMC4714868

[85] Antunes KH, Fachi JL, de Paula R, da Silva EF, Pral LP, dos Santos AÁ, Dias GBM, Vargas JE, Puga R, Mayer FQ, Maito F, Zárate-Bladés CR, Ajami NJ, Sant'Ana MR, Candreva T, Rodrigues HG, Schmiele M, Clerici S, Teresa Pedrosa M, Proença-Modena JL, Vieira AT, Mackay CR, Mansur D, Caballero MT, Marzec J, Li J, Wang X, Bell D, Polack FP, Kleeberger SR, Stein RT, Vinolo MAR, de Souza APD. 2019. Microbiota-derived acetate protects against respiratory syncytial virus infection through a GPR43-type 1 interferon response. Nat Commun 10:3273. doi:10.1038/s41467-019-11152-6. 31332169 PMC6646332

[86] Chakraborty K, Raundhal M, Chen BB, Morse C, Tyurina YY, Khare A, Oriss TB, Huff R, Lee JS, St Croix CM, Watkins S, Mallampalli RK, Kagan VE, Ray A, Ray P. 2017. The mito-DAMP cardiolipin blocks IL-10 production causing persistent inflammation during bacterial pneumonia. Nat Commun 8:13944. doi:10.1038/ncomms13944. 28074841 PMC5241690

[87] Galvão I, Tavares LP, Corrêa RO, Fachi JL, Rocha VM, Rungue M, Garcia CC, Cassali G, Ferreira CM, Martins FS, Oliveira SC, Mackay CR, Teixeira MM, Vinolo MAR, Vieira AT. 2018. The metabolic sensor GPR43 receptor plays a role in the control of Klebsiella pneumoniae infection in the lung. Front Immunol 9:142. doi:10.3389/fimmu.2018.00142. 29515566 PMC5826235

[88] Trompette A, Gollwitzer ES, Pattaroni C, Lopez-Mejia IC, Riva E, Pernot J, Ubags N, Fajas L, Nicod LP, Marsland BJ. 2018. Dietary fiber confers protection against flu by shaping Ly6c^−^ patrolling monocyte hematopoiesis and CD8^+^ T cell metabolism. Immunity 48:992–1005.e8. doi:10.1016/j.immuni.2018.04.022. 29768180

[89] National Research Council. 2011. Guide for the care and use of laboratory animals, 8th ed. National Academies Press, Washington, DC.

[90] Andersson B, Dahmén J, Frejd T, Leffler H, Magnusson G, Noori G, Edén CS. 1983. Identification of an active disaccharide unit of a glycoconjugate receptor for pneumococci attaching to human pharyngeal epithelial cells. J Exp Med 158:559–570. doi:10.1084/jem.158.2.559. 6886624 PMC2187347

[91] Andersson B, Eriksson B, Falsen E, Fogh A, Hanson LA, Nylén O, Peterson H, Svanborg Edén C. 1981. Adhesion of Streptococcus pneumoniae to human pharyngeal epithelial cells in vitro: differences in adhesive capacity among strains isolated from subjects with otitis media, septicemia, or meningitis or from healthy carriers. Infect Immun 32:311–317. doi:10.1128/iai.32.1.311-317.1981. 7216490 PMC350623

[92] Eisfeld AJ, Neumann G, Kawaoka Y. 2014. Influenza A virus isolation, culture and identification. Nat Protoc 9:2663–2681. doi:10.1038/nprot.2014.180. 25321410 PMC5619698

[93] Apprill A, McNally S, Parsons R, Weber L. 2015. Minor revision to V4 region SSU rRNA 806R gene primer greatly increases detection of SAR11 bacterioplankton. Aquat Microb Ecol 75:129–137. doi:10.3354/ame01753.

[94] Parada AE, Needham DM, Fuhrman JA. 2016. Every base matters: assessing small subunit rRNA primers for marine microbiomes with mock communities, time series and global field samples. Environ Microbiol 18:1403–1414. doi:10.1111/1462-2920.13023. 26271760

[95] R Core Team. 2018. R: a language and environment for statistical computing. R Foundation for Statistical Computing, Vienna, Austria. https://www.r-project.org/.

[96] Gierse LC, Meene A, Schultz D, Schwaiger T, Karte C, Schröder C, Wang H, Wünsche C, Methling K, Kreikemeyer B, Fuchs S, Bernhardt J, Becher D, Lalk M, KoInfekt SG, Urich T, Riedel K. 2020. A multi-omics protocol for swine feces to elucidate longitudinal dynamics in microbiome structure and function. Microorganisms 8:1887. doi:10.3390/microorganisms8121887. 33260576 PMC7760263

[97] Schiebenhoefer H, Schallert K, Renard BY, Trappe K, Schmid E, Benndorf D, Riedel K, Muth T, Fuchs S. 2020. A complete and flexible workflow for metaproteomics data analysis based on MetaProteomeAnalyzer and Prophane. Nat Protoc 15:3212–3239. doi:10.1038/s41596-020-0368-7. 32859984

[98] Tyanova S, Temu T, Sinitcyn P, Carlson A, Hein MY, Geiger T, Mann M, Cox J. 2016. The Perseus computational platform for comprehensive analysis of (prote)omics data. Nat Methods 13:731–740. doi:10.1038/nmeth.3901. 27348712

[99] Perez-Riverol Y, Bai J, Bandla C, García-Seisdedos D, Hewapathirana S, Kamatchinathan S, Kundu DJ, Prakash A, Frericks-Zipper A, Eisenacher M, Walzer M, Wang S, Brazma A, Vizcaíno JA. 2022. The PRIDE database resources in 2022: a hub for mass spectrometry-based proteomics evidences. Nucleic Acids Res 50:D543–D552. doi:10.1093/nar/gkab1038. 34723319 PMC8728295

