# Supplementary material for: Impact of Pneumococcal and Viral Pneumonia on the Respiratory and Intestinal Tract Microbiomes of Mice
Source: Microbiol Spectr. 2023 Mar 29;11(3):e03447-22. doi: 10.1128/spectrum.03447-22 (PMC10269894; doi:10.1128/spectrum.03447-22)
Supplement: Supplemental file 1 — Supplemental material. Download spectrum.03447-22-s0001.pdf, PDF file, 0.4 MB [file spectrum.03447-22-s0001.pdf]

Supplemental Material

Impact of pneumococcal and viral pneumonia on the respiratory and intestinal tract microbiomes of mice

Laurin Christopher Gierse[1], Alexander Meene[1], Sebastian Skorka[2], Fabian Cuypers[2], Surabhi Surabhi[2], Borja Ferrero-Bordera[1], Bernd Kreikemeyer[3], Dörte Becher[1], Sven Hammerschmidt[2]*, Nikolai Siemens[2], Tim Urich[1], and Katharina Riedel[1,*]

[1] Institute of Microbiology, University of Greifswald, Felix-Hausdorff-Str. 8, 17489 Greifswald, Germany; laurin.gierse@uni-greifswald.de (L.G.); alexander.meene@uni-greifswald.de (A.M.); borja.fererrobordera@uni-greifswald.de (B.F.); doerte.becher@uni-greifswald.de (D.B.), tim.urich@uni-greifswald.de (T.U.); riedela@uni-greifswald.de (K.R.)

[2] Department of Molecular Genetics and Infection Biology, Institute for Genetics and Functional Genomics, University of Greifswald, Felix-Hausdorff-Str. 8, 17489 Greifswald, Germany; Fabian.cuypers@uni-greifswald.de (F.C.); sven.hammerschmidt@uni-greifswald.de (S.H.); nikolai.siemens@uni-greifswald.de (N.S.)

[3] Institute for Medical Microbiology, Virology and Hygiene, Rostock University Medical Centre, Schillingallee 70, 18055 Rostock, Germany; bernd.kreikemeyer@med.uni-rostock.de

***** Correspondence: riedela@uni-greifswald.de (K.R.); Tel.: +493834-420-5900, sven.hammerschmidt@uni-greifswald.de (S.H.); Tel.: +493834-420-5700

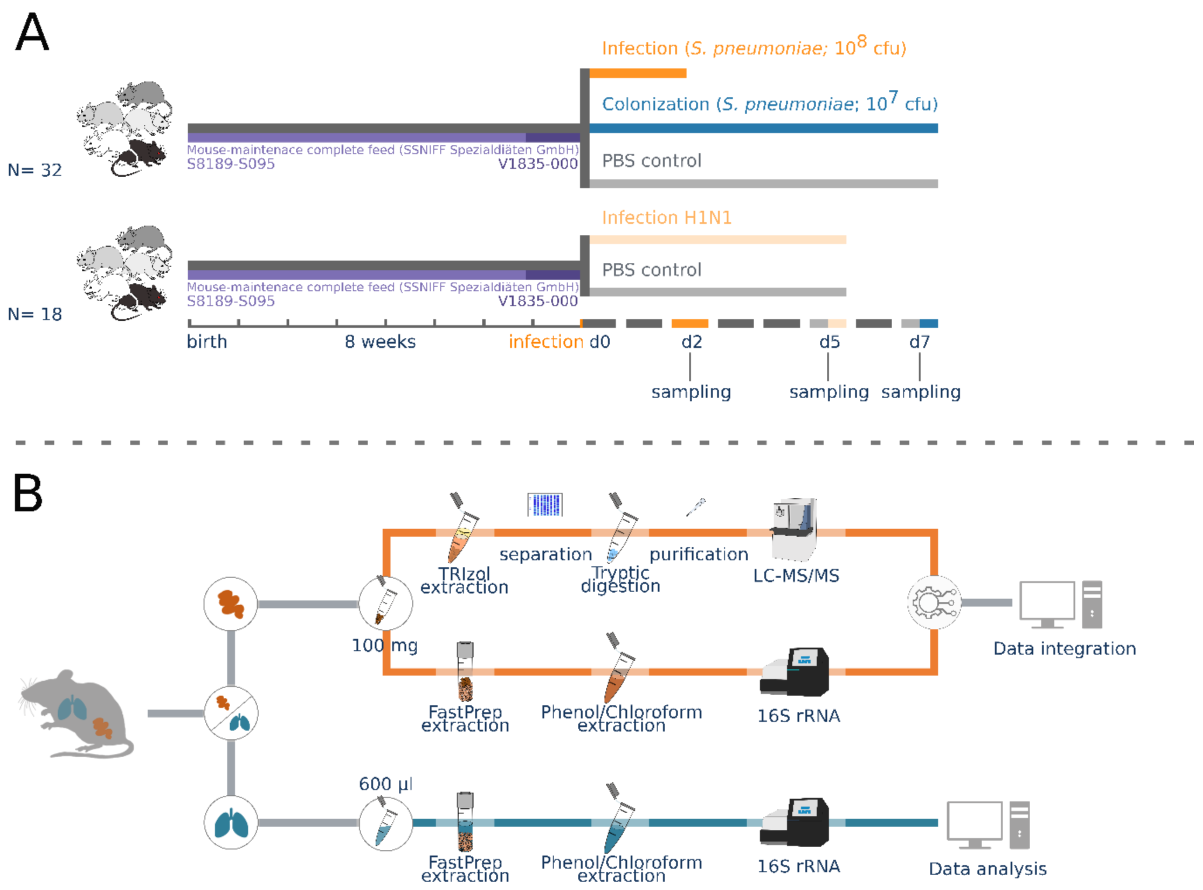

Figure S1: Experimental setup of the *S. pneumoniae* 19F and influenza A H1N1 infection trial (A). At the age of eight weeks, mice were colonized (N = 10) or infected (N = 12) with *S. pneumoniae*. A third group was treated with PBS and served as healthy control (N = 10). At day 2 after infection, severe pneumonia was observed in the mice infected with 10^8 CFU of S. pneumoniae. Mice were euthanized at day 2 (19F_P) and day 7 (19F_C and PBS). Nasal washes and cecal content was collected and stored on dry ice. In an independent experiment, one cohort was infected with influenza A (N = 12) and compared to a PBS treated control group (N = 6). Viral infected mice were euthanized at day 5 after infection and samples were taken. Subsequently, a meta-omics approach for cecal content and nasal washes obtained from C57BL/6J mice was applied (B). Briefly, mechanic cell disruption was performed before nucleic acid extraction using phenol/chloroform protocol. For protein extraction, a TRIzol based extraction protocol was used. After extraction, proteins were digested, purified and finally analyzed by massspectrometry.

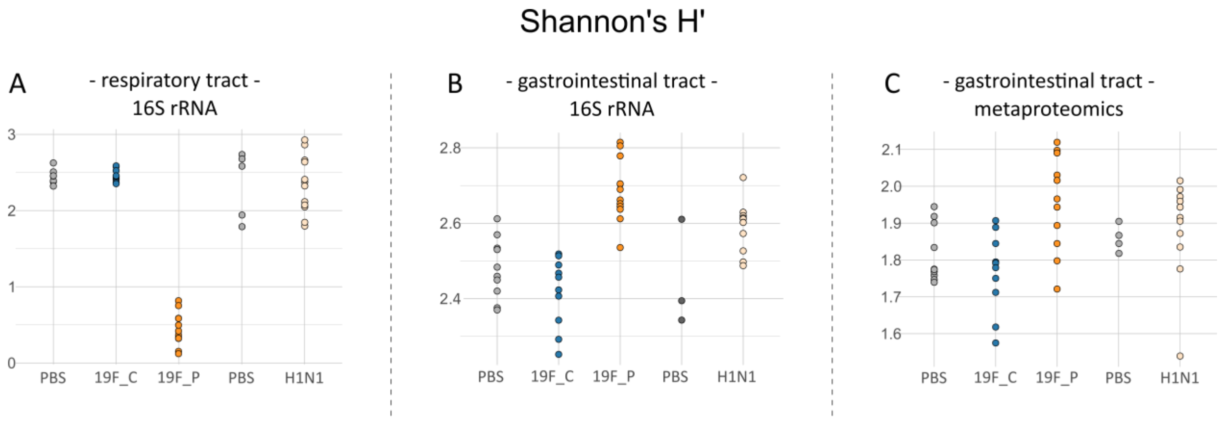

Figure S2: Shannon's H index based on the results of the taxonomic composition of the respiratory (A) and gastrointestinal tract (B & C) microbiome under healthy conditions and during pneumococcal colonization and infection, and influenza A infection. Each dot represents one animal. Number of mice analyzed in each group can be seen in **Table S1**.

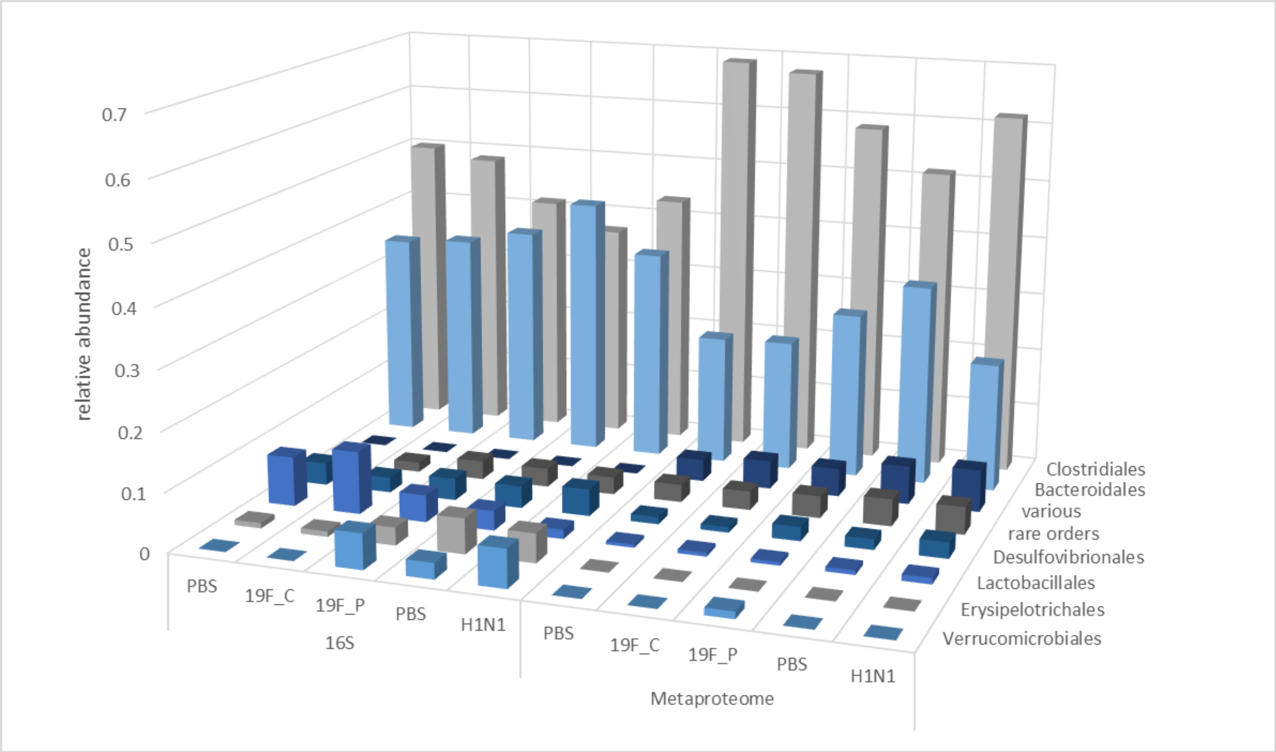

Figure S3: Taxonomic composition of the murine gastrointestinal microbiome on order level. For better illustration the six most abundant bacterial orders were shown. Orders below a relative abundance of 0.01 were grouped in the category rare orders. Identified protein groups assigned to multiple orders, based on the lowest common ancestor (LCA) algorithem, were assigned to the category various.

Table S1: Number of samples provided for meta-omics approach and number of analyzed samples using different meta-omics techniques. Deviations in the number of analyzed samples were due to limitations in biomass of individual samples.

		S. pneumoniae		Influenza A H1N1	
	PBS	colonized	infected	PBS	infected
Samples Provided (NALs)	12	10	10	6	12
16S rRNA gene sequencing	12	10	10	5	12
Metaproteomics	---	---	---	---	---

		S. pneumoniae		Influenza A H1N1	
	PBS	colonized	infected	PBS	infected
Samples Provided (Ceacum)	10	10	12	6	12
16S rRNA gene sequencing	9	10	12	3	11
Metaproteomics	10	10	11	4	12

Table S2: Average number of identified ASVs and PGs from 100 mg cecal content, also showing standard derivation and Shannon index.

	**PBS**	**19F_C**	**19F_P**	**PBS**	**H1N1**
**ASVs (NALs)**	317 (±39)	322 (±45)	27 (±6)	120 (±85)	165 (±65)
**Shannon's H**	2.43	2.45	0.42	2.31	2,32
**ASVs (caecum)**	1064 (±114)	1052 (±123)	461 (±60)	583 (±124)	640 (±101)
**Shannon's H**	2.47	2.41	2.68	2.45	2.59
**PGs (caecum)**	4375 (± 421)	4848 (±372)	6813 (± 199)	2838 (± 339)	4368 (± 717)
**Shannon's H**	1.81	1.77	1.95	1.85	1.88

Table S3: Person correlation of the taxonomic datasets from 16S rRNA gene sequencing and metaproteomic analysis. Comparison of the taxonomic profiles from the individual cohorts as well as experiment wide grouping are displayed.

**Pearson Correlation (16S vs. Metaproteome)**					
PBS	19F_C	19F_P	PBS	H1N1	over all
0.93	0.92	0.93	0.92	0.89	0.92
